# Cold tolerance of native plants in the Lancang River dry–hot valley: an integrative physiological–biochemical assessment with implications for cold-resistance breeding

**DOI:** 10.3389/fpls.2025.1724940

**Published:** 2026-01-27

**Authors:** Yunchen Zhang, Jianying Yang, Xu Yuan, Yandong Yang, Xiaodong Ji, Jinnan Ji, Yan Zhang, Jiao Huang

**Affiliations:** 1 College of Soil and Water Conservation, Beijing Forestry University, Beijing, China; 2 Huaneng Lancang River Hydropower Co., Ltd., Kunming, China; 3 Huaneng Lancang River Upstream Hydropower Co., Ltd., Changdu, China

**Keywords:** cold tolerance, cold-resistance breeding, Lancang River dry–hot valley, physiological–biochemical assessment, principal component analysis

## Abstract

**Introduction:**

Extreme winter cold in the Lancang River dry–hot valley limits vegetation establishment. Selecting cold-tolerant native species is therefore critical for ecological restoration and for maintaining stable agro-vegetation. This study aimed to assess cold tolerance in representative native shrubs and herbs using an integrated physiological and biochemical approach.

**Methods:**

Seedlings of three shrubs (*Sophora davidii*, *Vitex negundo* var. *microphylla*, *Rumex hastatus*) and two herbs (*Arthraxon lanceolatus*, *Artemisia vestita*) were exposed to temperatures from 25°C down to –35°C in growth chambers. We quantified membrane injury (relative electrolyte leakage and semilethal temperature, LT50), gas exchange, chlorophyll fluorescence, osmolyte levels (proline, soluble sugars), and antioxidant enzyme activities (e.g., superoxide dismutase). Multivariate analyses (principal component analysis and membership functions) were used to develop an integrated cold-tolerance index.

**Results:**

Semilethal temperature (LT50) differed markedly among species (approximately –27°C in *S. davidii* vs –5°C in *A. lanceolatus*), indicating a wide range of freezing tolerance. Across the freezing gradient, *S. davidii* maintained the lowest electrolyte leakage and partial Photosystem II efficiency, while accumulating high proline and soluble sugar levels and sharply increasing superoxide dismutase activity. In contrast, *A. lanceolatus* showed rapid membrane leakage and fluorescence declines. The most informative cold-response traits were Photosystem II efficiency and electrolyte leakage. An integrated cold-tolerance index based on multiple physiological metrics ranked species from highest to lowest tolerance as *A. vestita* > *A. lanceolatus* > *V. negundo* > *R. hastatus* > *S. davidii*. This ranking differed notably from the ranking based on LT50 alone.

**Discussion:**

The discrepancy between the multi-trait index and single-trait (LT50) ranking highlights the risk of inferring cold tolerance from one metric. Acute stress responses (membrane stability, photosynthesis) and long-term freezing thresholds capture complementary aspects of cold tolerance. The derived physiological thresholds and the multi-indicator framework provide practical guidance for selecting and breeding native species for ecological restoration and cold-resilient agriculture in dry–hot valleys.

## Graphical Abstract

**Figure:**
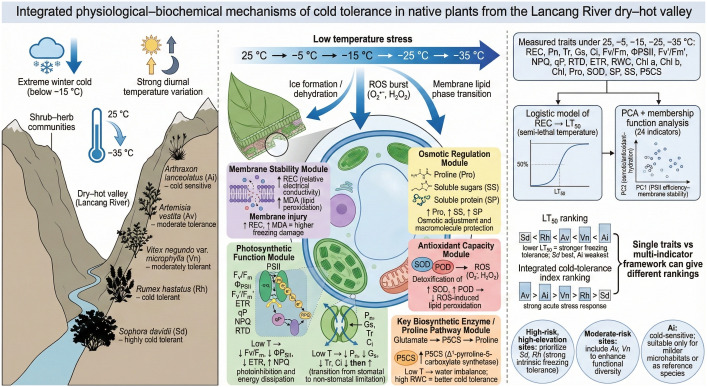
Experimental workflow of controlled low-temperature treatments and multi-indicator assessment of cold tolerance in five native species from the Lancang River dry–hot valley.

## 1 Introduction

The Lancang River dry–hot valley lies on the southeastern edge of the Tibetan Plateau, forming a deeply incised alpine gorge system. Its steep, paired topographic relief generates a striking vertical climatic gradient: warm, arid valley floors transition to cool, frigid mountaintops, fostering highly fragile ecosystems that are exceptionally sensitive to climatic fluctuations (Achard et al., 2006; Abdullaev et al., 2024). Winters regularly bring cold surges, with annual minimum temperatures often dropping below − 15°C and daily temperature ranges frequently exceeding 20°C. Precipitation is scarce and erratic, creating a persistent stress regime defined by the co-occurrence of chilling and aridity (Bajji et al., 2002; Apel and Hirt, 2004). As part of an ecologically vulnerable corridor in Southwest China and designated a Global Biosphere Hotspot—dominated by native shrubs and herbs—the dry–hot valley supports numerous highly adaptable species with substantial applied value (Baker, 2008; Chen and Li, 2016). Among these, three native shrubs (*Sophora davidii*, *Vitex negundo* var. *microphylla*, *Rumex hastatus*) and two native herbs (*Arthraxon lanceolatus*, *Artemisia vestita*) are broadly distributed. They form structurally critical components of the shrub–herb layer, playing key roles in ground cover, soil stabilization, and vegetation resilience along riverbanks and slopes. Their ecological prominence, broad environmental tolerance, and potential for restoration and agroecosystem applications make them ideal model taxa for investigating cold-stress responses in dry–hot valley native flora. Under ongoing climate warming, climatic extremes at plateau margins are intensifying; more frequent sudden cold snaps disrupt vegetation structure and function, underscoring the need to clarify cold-tolerance mechanisms across physiological and ecological scales and to develop robust evaluation frameworks for restoration practices (Chen and Wang, 2006; Chinnusamy et al., 2007).

Notably, dry–hot valley plants experience extreme daily temperature fluctuations within a complex topographic context, differing from taxa in humid regions. These conditions likely alter resource allocation between growth and stress defense, favoring unique cold-adaptation strategies (Demidchik et al., 2014; Didaran et al., 2024). Plant responses to low temperatures operate across multiple organizational levels (Du et al., 2019; Foyer and Noctor, 2020; Ding et al., 2024). At the cellular level, lipid peroxidation impairs membrane integrity, increasing relative electrical conductivity, while malondialdehyde serves as a reliable indicator of oxidative damage (Gill and Tuteja, 2010; Gao et al., 2019; Grebe et al., 2024). Functionally, cold stress compromises Photosystem II performance, reducing maximum quantum yield, effective quantum yield, and often electron transport rate, thereby restricting energy conversion and electron flow (IPCC, 2021; Henschel et al., 2024). Metabolically, accumulation of osmolytes (e.g., proline, soluble sugars) and activation of antioxidant enzymes (e.g., superoxide dismutase, peroxidase) mitigate osmotic stress and reactive oxygen species damage (Jiang et al., 2022; Jahed and Van den Ende, 2023). Molecularly, the C-repeat binding factor–cold-regulated (CBF-COR) network constitutes the core of cold acclimation (Jolliffe and Cadima, 2016). Despite these advances, to our knowledge, no quantitative, systematic analysis of cold tolerance exists for dry–hot valley shrubs and herbs that integrates multimetric physiological and photosynthetic data with defensible thresholds and region-specific evidence to guide species prioritization for restoration (Krause and Weis, 1991; Lantzouni et al., 2020; Körner, 2021; Li et al., 2023; Lauterberg et al., 2024).

To address this gap, we investigated five representative native shrub and herb species from the Lancang River dry–hot valley. These include three shrubs (*Sophora davidii*, *Vitex negundo* var. *microphylla*, *Rumex hastatus*) and two herbs (*Arthraxon lanceolatus*, *Artemisia vestita*). The selected species are either dominant or widely distributed in dry–hot valley shrub–herb communities, represent diverse life forms (N-fixing leguminous shrub, deciduous shrub, rhizomatous and tufted herbs, aromatic Asteraceae), and are already utilized or considered for ecological restoration, slope stabilization, and agroforestry in the region. They thus serve as both structural pillars of local vegetation and practical candidates or reference species for enhancing cold resistance in agricultural and restoration contexts.

We established an indicator system spanning five functional domains: (1) membrane stability (relative electrical conductivity and malondialdehyde), (2) gas-exchange capacity (net photosynthetic rate [Pn], transpiration rate [Tr], stomatal conductance [Gs], and intercellular CO_2_ concentration [Ci]), (3) chlorophyll fluorescence performance (maximum photochemical efficiency [*F_v_*/*F_m_*], maximum photochemical efficiency under light adaptation [*F_v_*′/*F_m_*′], actual photochemical efficiency of Photosystem II [ΦPSII], photochemical quenching coefficient [qP], nonphotochemical quenching [NPQ], and electron transport rate [ETR]), (4) osmotic and antioxidant regulation (proline, soluble sugars, soluble protein and superoxide dismutase [SOD] activity), and (5) proline biosynthesis (P5CS activity). These metrics were integrated using principal component analysis and membership-function approaches to derive a composite cold-tolerance index for species screening and restoration guidance. The region is recognized as a critical biodiversity hotspot (Zhang et al., 2011), yet ecosystem stability is threatened by increasing degradation (Liu et al., 2021) and the evolving characteristics of frost disasters in the river basin (Wang Y. et al., 2020). Understanding plant adaptation strategies in this context requires a fundamental analysis of frost survival mechanisms (Sakai and Larcher, 1987).

## 2 Materials and methods

### 2.1 Study area

Our field survey focused on the Deqin–Mangkang segment of the Lancang River (Diqing Tibetan Autonomous Prefecture, Yunnan Province, China; 98.049–98.830°E, 28.541–29.718°N), located within the Hengduan Mountains at the southeastern margin of the Tibetan Plateau. This segment forms a northeast–southwest trending valley system characterized by deep fluvial incision and pronounced topographic relief, typical of alpine-gorge landscapes. The survey spanned from Gushui northward along National Highway G214 (paralleling the Lancang River), encompassing the townships of Foshan, Muxu, and Quzika, and concluding in Rumei. Survey transects were designed to traverse valley floors, lower/upper slopes, and ridges, capturing a continuous topographic-ecological gradient. Originating in Zadoi County (Yushu Prefecture, Qinghai Province), the Lancang (Mekong) River flows ~ 4,880 km southward through six countries, ranking among Asia’s principal transboundary rivers. Near Deqin—adjacent to the high-elevation Kawagebo Peak (6,740 m a.s.l.)—extreme canyon incision and narrow valley bottoms form a geomorphic unit defined by fragmented terrain, active faulting, and steep slopes ranging from ~ 2,000 to 6,740 m. The region experiences a plateau–mountain monsoon climate, with a mean annual temperature of ~ 4.7°C, annual precipitation of 600–800 mm, and annual sunshine duration of ~ 1,981 h. Monsoon circulation interacting with complex terrain generates marked spatiotemporal variability in water availability (manifested as distinct wet and dry seasons), fostering substantial habitat heterogeneity and climatic sensitivity across vertical zonation and microtopographic gradients. These conditions shape distinct vegetation belts along the valley–hillslope–ridge gradient, driven by the combined effects of elevation, aspect, and water availability on plant community composition and structure. Arboreal communities are dominated by *Quercus aquifolioides* Rehder & E.H.Wilson, *Abies georgei* Orr, and *Picea likiangensis* (Franch.) E.Pritz, with associates including *Taxus wallichiana* and *Cupressus gigantea*. Shrub layers are typified by *Vitex negundo* var. *microphylla*, *Sophora davidii*, *Elsholtzia capituligera*, and *Ceratostigma minus* Stapf ex Prain, while common herbs include *Incarvillea arguta* Royle and *Selaginella tamariscina* (P. Beauv.) Spring. Collectively, these taxa form a transitional landscape—from evergreen/deciduous broadleaf and coniferous forests to shrub thickets and shrub–grass mosaics—along the elevational gradient.

### 2.2 Plant materials

In July–August 2024, we collected mature seeds of five dominant plant species, representing distinct families and growth habits, from 16 natural populations along the Deqin–Mangkang section of the Lancang River. The selected species included two herbs and three shrubs: (1) *Arthraxon lanceolatus* (Roxb.) Hochst. (Poaceae; Ai), a perennial herb reproducing by rhizomes that prefers moist, permeable soil; (2) *Artemisia vestita* Wall. ex Bess. (Asteraceae; Av), an aromatic herb; (3) *Rumex hastatus* D. Don (Polygonaceae; Rh), a highly adaptable shrub tolerant to drought, heat, and barren conditions, typically found on sandy slopes; (4) *Sophora davidii* Kom. ex Pavol. (Fabaceae; Sd), a heliophilous, drought-tolerant shrub with low soil requirements; and (5) *Vitex negundo* L. var. *microphylla* Hand.-Mazz. (Verbenaceae; Vn), a shrub or small tree commonly inhabiting riverside thickets at altitudes of 1,200–3,200 m.

Collections avoided strictly protected areas and followed the Yunnan Provincial Regulations on the Conservation of Wild Plant Resources. For each species, visibly insect−damaged or moldy seeds were discarded, and at least 500 healthy, well−filled seeds were retained per population.

### 2.3 Experimental design

#### 2.3.1 Treatments

Mature, fully expanded current−year leaves from three healthy plants per species were used for all measurements. After a 7−day preacclimation in a programmable growth chamber at 25°C/18°C (day/night), 60%–70% relative humidity, and a 14-h light/10-h dark photoperiod, plants were exposed to five temperature regimes: 25°C (control), − 5°C, − 15°C, − 25°C, and − 35°C.

For each species, leaves were pooled, divided into subsamples of equal fresh mass, and randomly assigned to the five temperature treatments. Each treatment comprised three biological replicates (individual plants), each with several technical replicates (leaves or leaf discs). Chamber temperature was decreased from 25°C to the target set point at 5°C h^−1^, held at the target temperature for 12 h, and then returned to 25°C at the same rate. All treatments were carried out under identical light and humidity conditions. Air temperature inside the chamber was monitored with a calibrated data logger.

At the end of each temperature treatment, leaf material for measurements of membrane permeability, gas exchange, and chlorophyll fluorescence was processed immediately. Samples for osmolyte, antioxidant enzyme, pigment, and enzyme assays were frozen in liquid nitrogen and stored at − 80°C until analysis.

#### 2.3.2 Determination of semilethal temperature under cold stress

Cold-induced membrane injury was quantified as relative electrical conductivity (REC, %). For each species, mean REC values were computed from three biological replicates × four technical replicates at each temperature (25°C control; − 5°C, − 15°C, − 25°C, and − 35°C) and used for model fitting (Equation 1) (Liu, 2000):

(1) $$\text{y}=\frac{100}{1+{\text{ae}}^{-\text{bx}}}$$

Where *y* is REC (%), *x* is stress temperature (°C), and *a* and *b* are parameters estimated under constraints *a* > 0, *b* > 0. Empirical initial values were supplied to facilitate convergence. When heteroscedasticity across temperatures was evident, weighted fitting was applied with *w*=1/*s*^2^ (sample standard deviation [*s*]).

The semilethal temperature was defined at *y*=50% (Equation 2):

(2) $${\text{LT}}_{50}=\frac{\text{lna}}{\text{b}}$$

Uncertainty (95% confidence interval [CI]) was propagated from the fitted covariance matrix. The relationship between relative electrical conductivity and temperature was fitted using the logistic equation as established by Zhu et al. (1986): y = K / (1 + ae^-bx^) (Equation 1). The semilethal temperature (LT50) was calculated as the inflection point of the curve, following analytical protocols reviewed by Zhu (2016).

#### 2.3.3 Relative electrical conductivity under cold stress

Cold−induced membrane injury was quantified as relative electrical conductivity. For each species × temperature combination, three biological replicates were prepared; each replicate consisted of four tubes containing approximately 0.20 g of leaf tissue (midribs removed). Leaf segments were rinsed with deionized water, blotted dry, and immersed in 30 mL of deionized water. After 12 h at 25°C, initial conductivity (*C*_0_) was measured. The same tubes were then heated to 98°C for 1.5 h to release all electrolytes, cooled to room temperature, and equilibrated at 25°C for 10 h before the final conductivity (*C_t_*) was recorded. Relative conductivity (%) was calculated as (*C*_0_ − *C_b_*)/(*C_t_* − *C_b_*) × 100, where *C_b_* is the blank value for deionized water (Liu et al., 2018). Higher values indicate greater membrane damage.

#### 2.3.4 Gas-exchange measurements under cold stress

Leaf gas exchange was measured immediately after temperature treatments using a portable photosynthesis system (GFS−3000; Heinz Walz GmbH, Effeltrich, Germany). Net photosynthetic rate, transpiration rate, stomatal conductance, and intercellular CO_2_ concentration were recorded on three to five fully expanded leaves per plant within a fixed time window (09:00–11:00 hours). During measurements, plants were equilibrated for 30 min at 25°C and 60%–70% relative humidity.

Leaf−chamber conditions were standardized (photosynthetic photon flux density: 1,800 µmol m^−2^ s^−1^, reference CO_2_ concentration: 400 µmol mol^−1^, flow rate: 700 µmol s^−1^). For each leaf, readings were taken after a steady state had been reached and averaged across technical replicates. Values were then averaged across three biological replicates per species and temperature.

#### 2.3.5 Chlorophyll fluorescence imaging under cold stress

Chlorophyll fluorescence imaging was performed using an Imaging-PAM system (MAXI version; Heinz Walz GmbH, Effeltrich, Germany). Measurements were conducted in accordance with the standard protocols described by Krause and Weis (1991) and Maxwell and Johnson (2000), with minor modifications. Briefly, measurements were conducted immediately after each temperature treatment: leaves were first equilibrated for 30 min at 25°C and 60%–70% relative humidity, then dark-adapted for 20 min to determine initial fluorescence (*F*_0_) and maximum fluorescence (*F_m_*). After actinic illumination, maximum fluorescence under light adaptation (*F_m_*′) and initial fluorescence under light adaptation (*F*_0_′) were recorded. Subsequently, *F_v_*/*F_m_*, ΦPSII, qP, *F_v_*′/*F_m_*′, NPQ, and ETR were calculated using the instrument software. For statistical analysis, three plants per species were measured under each temperature treatment, and the values were averaged.

#### 2.3.6 Physiological and biochemical measurements under cold stress

##### 2.3.6.1 Leaf relative water content

Leaf relative water content was determined immediately after cold exposure. For each species and temperature, three biological replicates were collected (three leaves per replicate). Fresh mass was recorded, leaves were rehydrated in deionized water at 4°C for 20–24 h to obtain turgid mass, and dry mass was measured after oven−drying at 80°C to constant weight. Relative water content (%) was calculated as Equation 3:

(3) $$\text{RWC}(\%)=\frac{\text{FW}-\text{DW}}{\text{TW}-\text{DW}}\times 100$$

##### 2.3.6.2 Cold-tolerance physiological/biochemical indices

Mature leaves were sampled immediately after temperature treatment for osmolyte and antioxidant measurements. Free proline was quantified by the acidic ninhydrin method (Bates, 1973). Soluble sugars and soluble protein were determined using established colorimetric assays (Yemm and Willis, 1954; Bradford, 1976). Superoxide dismutase activity was assayed via inhibition of nitroblue tetrazolium reduction under light (Giannopolitis and Ries, 1977). Unless otherwise stated, all indices are expressed on a fresh-mass basis as mean ± standard deviation.

##### 2.3.6.3 Chlorophyll content

Chlorophyll *a*, chlorophyll *b*, and total chlorophyll were determined following Ma et al. (2020). Briefly, 0.20 g of fresh leaf tissue was extracted with 95% ethanol in the dark until fully decolorized, and absorbance at 665 and 649 nm was measured to calculate pigment concentrations, expressed on a fresh-mass basis.

##### 2.3.6.4 Proline biosynthetic enzyme activity

Activity of Δ^1^−pyrroline−5−carboxylate synthetase, a key enzyme in proline biosynthesis, was measured in crude leaf extracts prepared in Tris–HCl buffer (pH 8.0) containing ethylenediaminetetraacetic acid, MgCl_2_, and dithiothreitol. Nicotinamide adenine dinucleotide phosphate (NADPH) oxidation at 340 nm was monitored at 37°C in reaction mixtures containing glutamate, ATP, and MgCl_2_. Enzyme activity was calculated from the linear decrease in absorbance using the molar extinction coefficient of NADPH and expressed as international units per gram fresh mass.

#### 2.3.7 Membership-function-based assessment of cold tolerance

Following the standardization framework (Ma et al., 2021), we constructed a cold-tolerance evaluation matrix using species-level means for each temperature treatment (− 5°C, − 15°C, − 25°C, − 35°C; three biological replicates with ≥ 3 technical replicates per biological replicate). The indicator suite comprised physiological, photosynthetic, and biochemical variables, including REC, Pn, Tr, Gs, Ci, *F*_0_, *F_m_*, *F_v_*, *F_v_*/*F_m_*, *F_v_*′/*F_m_*′, ΦPSII, NPQ, qP, RTD, ETR, RWC, Chl *a*, Chl *b*, Chl, Pro, SOD, SP, SS, and proline biosynthetic enzyme activity.

For each indicator *k*, the membership degree *H_ik_* of species iii was computed as:

Positively associated indicators (higher value → greater cold tolerance) (Equation 4):

(4) $$H_{ik}=\frac{X_{ik}-X_{k}^{\text{min}}}{X_{k}^{\text{max}}-X_{k}^{\text{min}}}$$

Negatively associated indicators (higher value → lower cold tolerance) (Equation 5):

(5) $$H_{ik}=1-\frac{X_{ik}-X_{k}^{\text{min}}}{X_{k}^{\text{max}}-X_{k}^{\text{min}}}$$

Where ${\text{X}}_{ik}$ is the observed mean of species i, and $X_{k}^{min},X_{k}^{max}$ are the minimum and maximum values across all species. If $X_{k}^{\text{max}}=X_{k}^{\text{min}}$, we set $H_{ik}=0.5$ to avoid division by zero. The membership matrices $\left(H_{ik}\right)$ were subjected to principal component analysis (PCA) after passing the KMO and Bartlett’s tests. Components with eigenvalues >1 were retained. Indicator weights ${\text{w}}_{\text{k}}$ were derived from component loadings $\left(l_{kp}\right)$ and variance contributions $\left(\gamma_{p}\right)$ as Equation 6:

(6) $$w_{k}=\frac{\sum_{p=1}^{m}\gamma_{p}|l_{kp}|}{\sum_{k=1}^{K}\sum_{p=1}^{m}\gamma_{p}|l_{kp}|},$$

followed by normalization to ensure

$\sum_{k=1}^{K}{\text{w}}_{\text{k}}=1$. The composite cold-tolerance index for species

i was then calculated as Equation 7:

(7) $$CI_{i}=\sum_{k=1}^{K}w_{k}H_{ik},$$

with larger $CI_{i}$ values indicating stronger cold tolerance. Species were ranked and assigned to tolerance classes based on the CI distribution, following the criteria of Maxwell and Johnson (2000) and Mattila et al. (2020).

### 2.4 Data processing and statistical analysis

All variables are reported as mean ± standard deviation (*n*=3). Normality and homogeneity of variance were checked before analysis. One-way ANOVA, followed by appropriate *post-hoc* comparisons (Tukey), was used to assess the effects of temperature and species (*p*<0.05). Semilethal temperature (LT_50_) was estimated by logistic fitting. Analyses were performed in OriginPro 2024 and IBM SPSS Statistics 22.0.

## 3 Results and analysis

### 3.1 Semilethal temperature and membrane injury responses to low-temperature stress

Semilethal temperatures calculated using three-parameter logistic regression revealed significant interspecific differences among native dry–hot valley species (**Supplementary Table S1**). Since semilethal temperatures serve as an inverse indicator of cold tolerance, *Sophora davidii* exhibited the strongest freezing tolerance (corresponding to the lowest semilethal temperatures), followed by *Rumex hastatus*, *Artemisia vestita*, and *Vitex negundo* var. *microphylla*. *Arthraxon lanceolatus* showed the weakest cold tolerance, corresponding to the highest semilethal temperatures. Logistic models effectively described the nonlinear cold injury responses across all species, with coefficients of determination (*R*^2^) ranging from 0.56 to 0.69. These results confirm that the fitted parameters reliably captured the progression of cold-induced damage throughout the tested temperature gradient.

**Figure 1:**
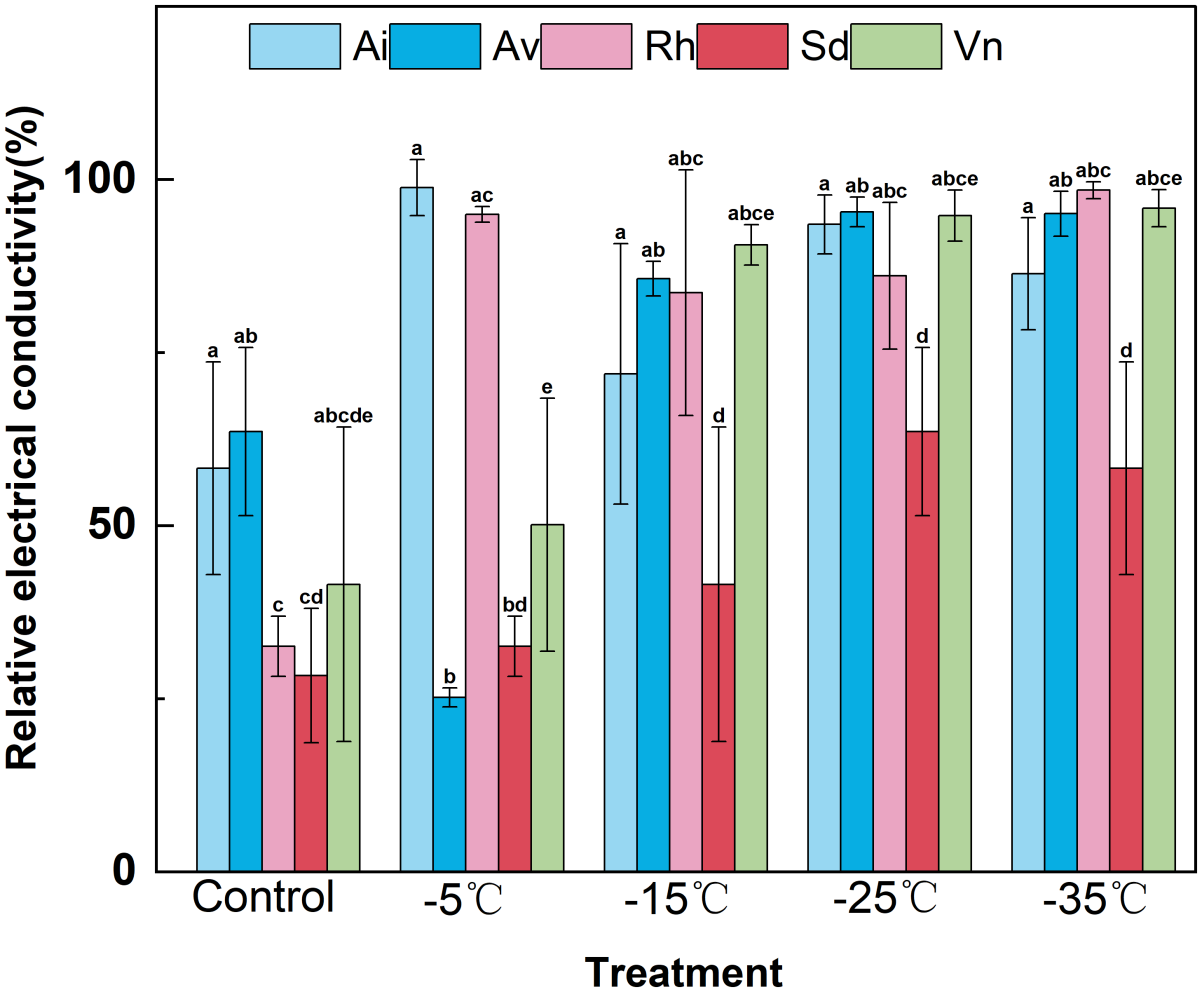
Effects of low-temperature stress on the relative conductivity of different plant species. Data are means ± SD (*n*=3). Different lowercase letters above the bars indicate significant differences among species at the same temperature (one−way ANOVA, Duncan’s test, *p*<0.05).

Relative electrical conductivity patterns closely matched the cold-tolerance rankings derived from semilethal temperatures (**Figure 1**). Relative electrical conductivity increased significantly as temperature decreased across all species (*p*<0.05); notably, *Sophora davidii* maintained the lowest electrolyte leakage at every tested temperature, indicating superior membrane stability. In contrast, *Rumex hastatus* and *Arthraxon lanceolatus* consistently exhibited the highest relative electrical conductivity values across the temperature gradient, while *Artemisia vestita* and *Vitex negundo* var. *microphylla* showed intermediate levels of membrane injury. Considering the full temperature range, the overall sensitivity of membrane damage—as inferred from relative electrical conductivity—followed the order: *Rumex hastatus* ≈ *Arthraxon lanceolatus* > *Artemisia vestita* ≈ *Vitex negundo* var. *microphylla* > *Sophora davidii*. Collectively, semilethal temperature estimates and relative electrical conductivity responses confirm that *Sophora davidii* exhibits the strongest cold resilience at the cellular level, whereas *Arthraxon lanceolatus* and *Rumex hastatus* are most vulnerable to cold-induced membrane injury under the winter conditions typical of the Lancang River dry–hot valley.

### 3.2 Effects of low-temperature stress on photosynthetic parameters

**Figure 2:**
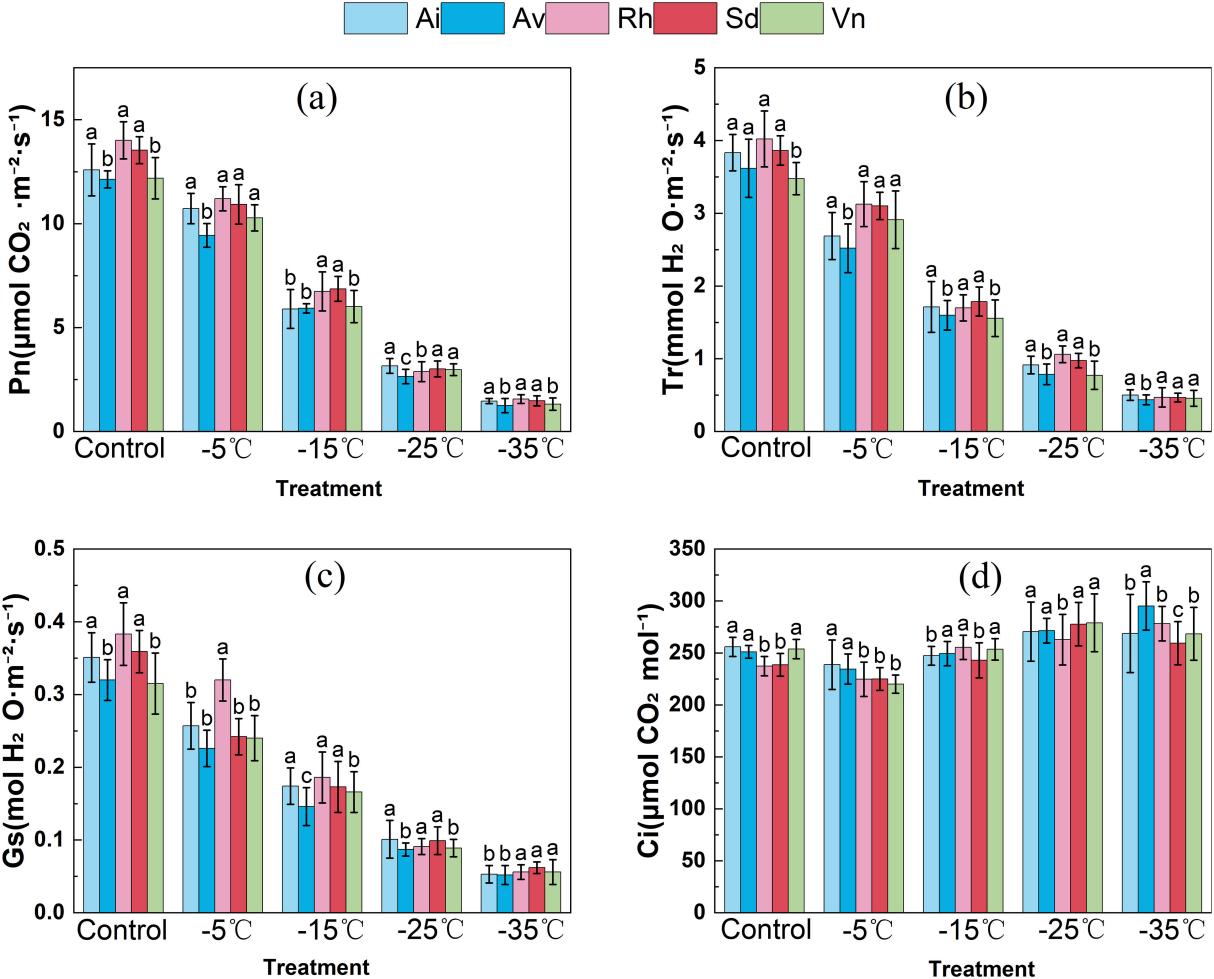
**(A)** Net photosynthetic rate; **(B)** Stomatal conductance; **(C)** Intercellular CO_2_; **(D)** Transpiration rate for five species under control and freezing temperatures. Effects of low−temperature stress on gas−exchange parameters in five native species. Data are means ± SD (*n*=3). Different lowercase letters above the bars indicate significant differences among species at the same temperature (one−way ANOVA, Duncan’s test, *p*<0.05).

Low-temperature exposure significantly altered leaf gas-exchange traits (**Figure 2**), with both temperature and species identity exerting significant effects on all measured parameters, and distinct interspecific differences observed at each temperature. At 25°C, *Arthraxon lanceolatus* and *Artemisia vestita* had the highest net photosynthetic rates (approximately 12 and 11 µmol CO_2_ m^−2^ s^−1^), transpiration rates (approximately 4–5 mmol H_2_O m^−2^ s^−1^), and stomatal conductances (approximately 0.3–0.4 mol H_2_O m^−2^ s^−1^), whereas *Rumex hastatus*, *Sophora davidii*, and *Vitex negundo* var. *microphylla* showed lower values (net photosynthetic rates approximately 8–10 µmol CO_2_ m^−2^ s^−1^; transpiration rates and stomatal conductances significantly lower). All three parameters declined with cooling, especially below − 15°C. Under severe cold (− 25°C to − 35°C), *Sophora davidii* (and secondarily *Rumex hastatus*) retained a higher proportion of their control net photosynthetic rate (for example, *Sophora davidii* retained approximately 25% vs. *Arthraxon lanceolatus*, which retained approximately 15% at − 35°C), transpiration rate, and stomatal conductance than *Arthraxon lanceolatus* and *Artemisia vestita*, indicating superior carbon assimilation capacity and stomatal regulation ability.

Intercellular CO_2_ concentration exhibited a characteristic “decrease–then–increase” pattern (**Figure 2D**). From 25°C to − 15°C, intercellular CO_2_ concentration decreased significantly in all species (for example, *Arthraxon lanceolatus* declined from approximately 250 to 200 µmol CO_2_ mol^−1^), reflecting stomatal closure as the primary limitation to photosynthesis. However, between − 25°C and − 35°C, intercellular CO_2_ concentration rebounded (for example, *Sophora davidii* reached ≥ 250 µmol CO_2_ mol^−1^) while net photosynthetic rate continued to decline—a typical signature of emerging nonstomatal limitations (e.g., biochemical or photochemical inefficiency) as the photosynthetic apparatus deteriorated despite reduced stomatal conductance.

The species-specific trajectories of these gas-exchange parameters reflect interspecific differences in both stomatal regulation and the intrinsic cold stability of the photosynthetic machinery. Under extreme cold, *Sophora davidii* and *Rumex hastatus* more effectively coordinate gas exchange with carbon fixation than *Arthraxon lanceolatus* and *Artemisia vestita*, consistent with their higher cold resilience inferred from relative electrical conductivity and semilethal temperature rankings.

### 3.3 Effects of low-temperature stress on chlorophyll fluorescence parameters

Ten chlorophyll fluorescence indices changed systematically with decreasing temperature, and significant species-specific differences were observed at each cold exposure level (**Table 1**). A detailed analysis of all indicators is provided in **Supplementary Table S2**, which lists the most representative indicators. For basal fluorescence, maximal fluorescence, and variable fluorescence—where variable fluorescence is defined as the difference between maximal fluorescence and basal fluorescence—interspecific variations were evident at 25°C: *Artemisia vestita* had the highest basal fluorescence (301.5 ± 20.9), while *Sophora davidii* exhibited the highest maximal fluorescence (1,206.7 ± 72.0) and variable fluorescence (0.536 ± 0.037). Under − 35°C, *Sophora davidii* showed the minimal elevation in basal fluorescence (468.3 ± 23.6) and retained the highest maximal fluorescence (797.7 ± 42.0) and variable fluorescence (0.263 ± 0.014) compared with other species, indicating less damage to the Photosystem II antenna system and preserved photochemical potential. In contrast, *Rumex hastatus* had a sharp increase in basal fluorescence (450.7 ± 39.1), reflecting stronger excitation pressure on Photosystem II.

**Table 1:** Effects of low-temperature stress on chlorophyll fluorescence parameters in different plant species.

Temperature (°C)	Species	Chlorophyll fluorescence parameter index			
		*F_v_*/*F_m_*	ΦPSII	NPQ	ETR
25	AI	0.744 ± 0.022 a	0.371 ± 0.04 a	0.98 ± 0.15 b	78.1 ± 8.9 a
25	Av	0.733 ± 0.021 b	0.321 ± 0.049 b	1.05 ± 0.08 a	66.3 ± 10.8 b
25	Rh	0.769 ± 0.02 a	0.398 ± 0.036 a	0.91 ± 0.09 b	81.7 ± 8.4 a
25	Sd	0.758 ± 0.026 a	0.42 ± 0.038 a	0.87 ± 0.09 c	86.7 ± 7.5 a
25	Vn	0.739 ± 0.012 a	0.365 ± 0.034 a	0.88 ± 0.12 c	77.0 ± 4.7 a
− 5	AI	0.706 ± 0.022 c	0.305 ± 0.029 b	1.25 ± 0.15 a	63.1 ± 6.5 b
− 5	Av	0.71 ± 0.027 b	0.298 ± 0.031 b	1.29 ± 0.11 a	64.2 ± 7.0 b
− 5	Rh	0.721 ± 0.032 b	0.339 ± 0.052 a	1.25 ± 0.11 a	70.7 ± 9.1 a
− 5	Sd	0.736 ± 0.015 a	0.339 ± 0.03 a	1.22 ± 0.1 b	71.2 ± 7.2 a
− 5	Vn	0.71 ± 0.011 b	0.307 ± 0.054 b	1.3 ± 0.09 a	66.3 ± 14.7 a
− 15	AI	0.626 ± 0.016 b	0.239 ± 0.017 a	1.87 ± 0.08 b	50.2 ± 5.4 a
− 15	Av	0.634 ± 0.032 b	0.204 ± 0.03 a	1.83 ± 0.12 b	40.3 ± 6.7 b
− 15	Rh	0.657 ± 0.019 a	0.248 ± 0.02 a	1.88 ± 0.14 b	53.2 ± 3.7 a
− 15	Sd	0.637 ± 0.022 b	0.234 ± 0.034 a	1.9 ± 0.11 a	50.8 ± 6.2 a
− 15	Vn	0.619 ± 0.029 c	0.196 ± 0.029 b	1.95 ± 0.13 a	42.1 ± 5.8 b
− 25	AI	0.539 ± 0.044 b	0.144 ± 0.012 a	2.63 ± 0.1 a	30.0 ± 2.9 a
− 25	Av	0.534 ± 0.045 b	0.116 ± 0.019 b	2.66 ± 0.09 a	25.3 ± 4.3 b
− 25	Rh	0.569 ± 0.028 a	0.134 ± 0.016 a	2.68 ± 0.08 a	28.9 ± 5.2 a
− 25	Sd	0.579 ± 0.028 a	0.138 ± 0.016 a	2.57 ± 0.08 b	29.2 ± 2.8 a
− 25	Vn	0.518 ± 0.04 b	0.129 ± 0.007 a	2.62 ± 0.09 b	26.5 ± 1.8 b
− 35	AI	0.434 ± 0.026 a	0.079 ± 0.009 b	3.37 ± 0.08 a	17.3 ± 3.9 a
− 35	Av	0.399 ± 0.042 a	0.086 ± 0.018 a	3.35 ± 0.09 a	17.6 ± 4.7 a
− 35	Rh	0.465 ± 0.057 a	0.092 ± 0.013 a	3.35 ± 0.1 a	17.1 ± 2.5 a
− 35	Sd	0.41 ± 0.063 a	0.09 ± 0.014 a	3.35 ± 0.1 a	18.3 ± 3.7 a
− 35	Vn	0.358 ± 0.052 b	0.088 ± 0.023 a	3.36 ± 0.15 a	17.1 ± 5.9 a

Plant abbreviations used in the figures are as follows: Ai, Arthraxon lanceolatus; Av, Artemisia vestita; Rh, Rumex hastatus; Sd, Sophora davidii; Vn, Vitex negundo var. microphylla.

All results in this table are expressed as mean ± standard deviation. Different lowercase letters (a–c) indicate significant differences among species at the same temperature (one-way analysis of variance [ANOVA] followed by Duncan’s multiple range test, p<0.05).

Photochemical efficiency parameters—including the maximum quantum yield of Photosystem II, the effective quantum yield of Photosystem II, and actual quantum yield of Photosystem II—and electron transport rate further highlighted *Sophora davidii*’s cold tolerance. At 25°C, all species had ideal values of the maximum quantum yield of Photosystem II (ranging from 0.733 to 0.769), with *Sophora davidii* also showing a higher effective quantum yield of Photosystem II (0.536 ± 0.027) and actual quantum yield of Photosystem II (0.420 ± 0.028) than *Arthraxon lanceolatus*. Although the maximum quantum yield of Photosystem II decreased significantly at − 35°C, the absolute decline relative to the control was smaller for *Sophora davidii* (final value: 0.410 ± 0.063) than for *Arthraxon lanceolatus* (final value: 0.434 ± 0.026), suggesting better protection of Photosystem II reaction centers in *Sophora davidii*. In addition, *Sophora davidii* maintained a higher electron transport rate at 25°C (86.7 ± 7.5) and sustained substantial electron flux at − 35°C (18.3 ± 3.7), a level comparable to or slightly higher than that of *Arthraxon lanceolatus*, indicating superior stability of the electron transport chain.

Energy allocation parameters, including nonphotochemical quenching, photochemical quenching, and regulated thermal dissipation, revealed *Sophora davidii’s* optimized photoprotection mechanisms under cold stress. Nonphotochemical quenching increased with cooling, and *Arthraxon lanceolatus* and *Sophora davidii* exhibited similar nonphotochemical quenching values (approximately 3.3) at − 35°C. However, *Sophora davidii* sustained a stable actual quantum yield of Photosystem II despite this elevated heat dissipation. *Sophora davidii* maintained higher photochemical quenching (an indicator of open Photosystem II centers) at both 25°C (0.767 ± 0.059) and − 35°C (0.355 ± 0.046) compared with *Arthraxon lanceolatus*. At − 35°C, the regulated thermal dissipation of *Sophora davidii* approached that of *Arthraxon lanceolatus* (0.752 ± 0.046 vs. 0.774 ± 0.050). Collectively, *Sophora davidii* and secondarily *Rumex hastatus* preserved Photosystem II structure and function by balancing photochemical energy utilization and regulating heat loss, whereas *Arthraxon lanceolatus* and *Artemisia vestita* exhibited earlier and more pronounced declines in fluorescence parameters under low-temperature conditions.

### 3.4 Effects of low-temperature stress on physiological and biochemical indices

#### 3.4.1 Relative water content

**Figure 3:**
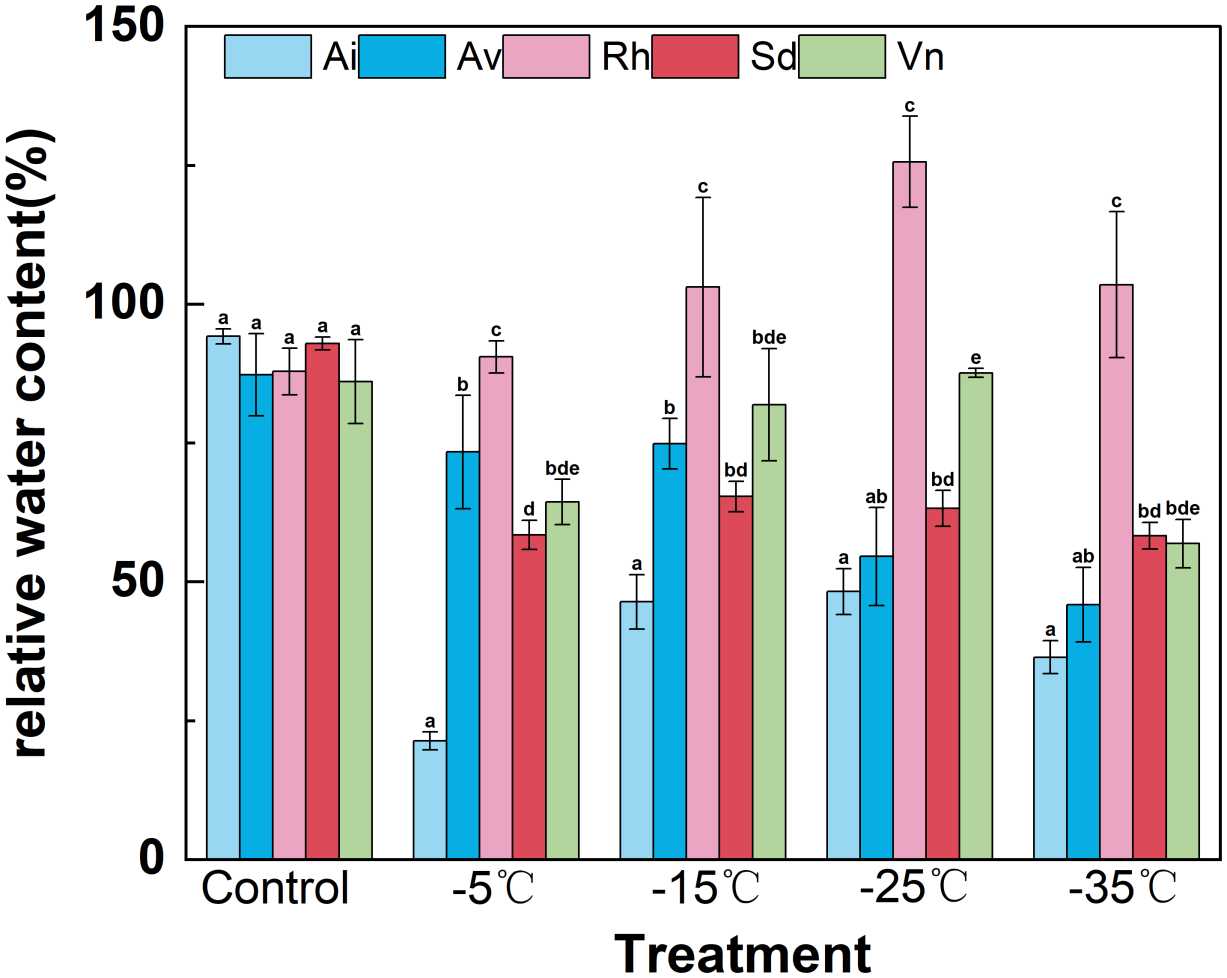
Effects of low−temperature stress on leaf relative water content (RWC). Data are means ± SD (*n*=3). Different lowercase letters above the bars indicate significant differences among species at the same temperature (one−way ANOVA, Duncan’s test, *p*<0.05).

Relative water content showed significant species-specific variations and distinct temperature-dependent responses among the studied species (**Figure 3**). Under control conditions (25°C), interspecific differences in relative water content were modest: *Arthraxon lanceolatus* maintained the highest relative water content (approximately 95%), *Rumex hastatus* and *Sophora davidii* retained approximately 90%, whereas *Artemisia vestita* and *Vitex negundo* var. *microphylla* exhibited the lowest baseline hydration (approximately 85%), indicating generally comparable leaf water status at ambient temperature.

With progressive cooling from − 5°C to − 35°C, relative water content declined in most species; however, *Rumex hastatus* consistently maintained a significantly higher water status across the entire low-temperature gradient. Specifically, *Rumex hastatus* retained approximately 90% relative water content at − 5°C (vs. only approximately 25% in *Arthraxon lanceolatus*), maintained approximately 105% at − 15°C (vs. approximately 45% in *Arthraxon lanceolatus*), and peaked at approximately 130% at − 25°C. At this temperature, *Sophora davidii* and *Vitex negundo* var. *microphylla* reached approximately 65%, whereas *Artemisia vestita* and *Arthraxon lanceolatus* reached approximately 50%. Notably, *Rumex hastatus* remained at approximately 105% relative water content even at − 35°C (compared with approximately 60% in *Sophora davidii* and *Vitex negundo* var. *microphylla* and approximately 35% in *Arthraxon lanceolatus*). In contrast, *Arthraxon lanceolatus* exhibited a pronounced, temperature-dependent decline in relative water content throughout the cooling process.

Collectively, *Rumex hastatus* exhibited the minimal net reduction in relative water content across temperatures, consistent with its efficient alleviation of cold-induced dehydration—likely mediated by osmotic adjustment and preserved membrane integrity. This robust water-retention capacity represents a key adaptive trait underpinning *Rumex hastatus’s* tolerance to winter chilling. In contrast, *Arthraxon lanceolatus’s* rapid decline in relative water content reflects high vulnerability to cold-induced water imbalance. These interspecific variations in leaf water status constitute a distinct physiological hallmark of divergent cold tolerance among native species of the Lancang River dry–hot valley, highlighting the ecological significance of water retention for cold adaptation in this harsh environment.

#### 3.4.2 Osmotic regulation and antioxidant responses

**Figure 4:**
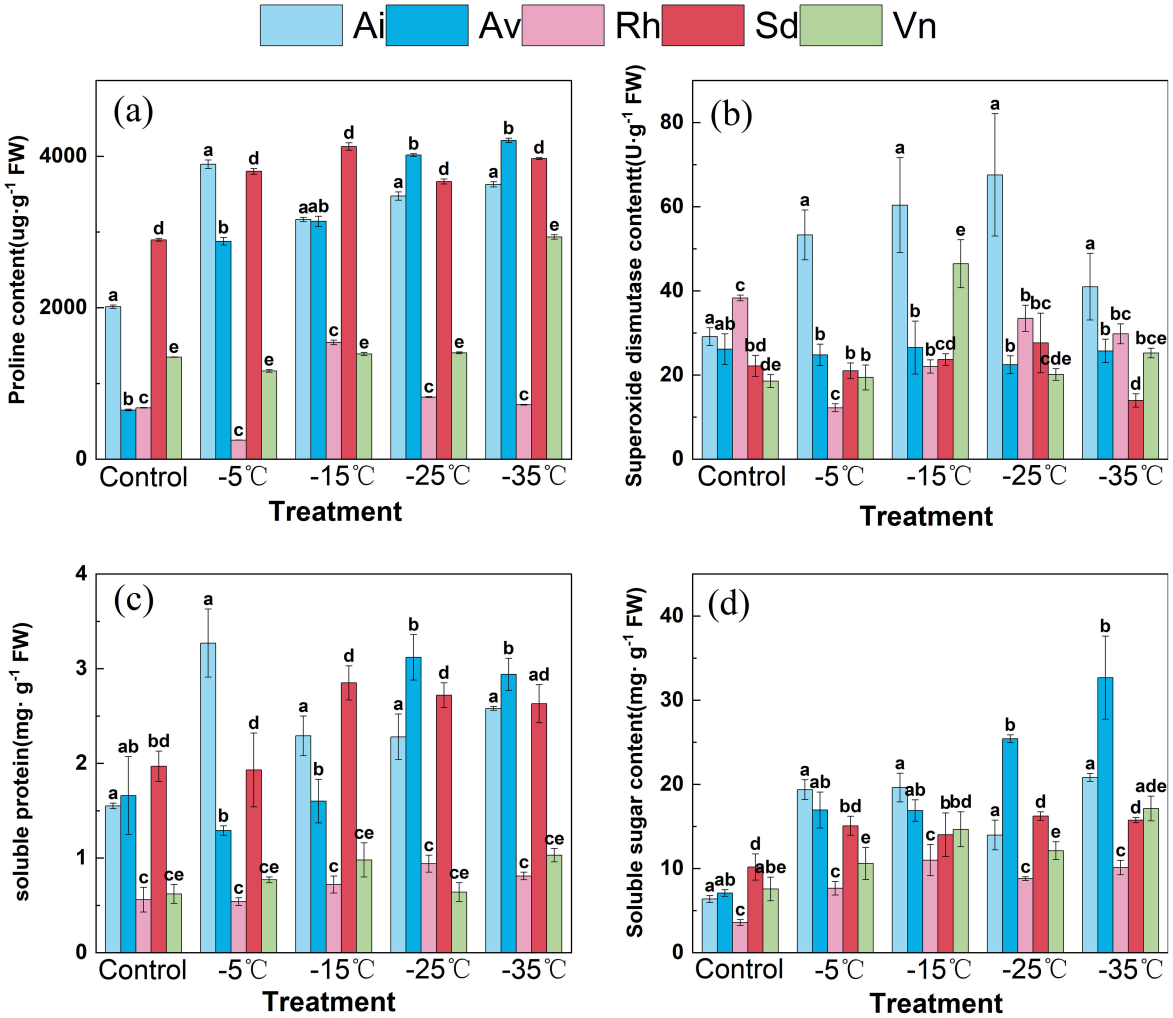
**(A)** Proline content; **(B)** SOD activity; **(C)** Soluble protein; **(D)** Soluble sugar in the five species at various temperatures. Effects of low−temperature stress on osmolytes and antioxidant activity in leaves of five native species. Data are means ± SD (*n*=3). Different lowercase letters above the bars indicate significant differences among species at the same temperature (one−way ANOVA, Duncan’s test, *p*<0.05).

Osmotic adjustment metabolites (proline, soluble protein, soluble sugars) and superoxide dismutase activity showed significant interspecific variations, with consistent temperature-dependent responses across gradients (**Figure 4**). Among the five species, the cold-tolerant *Sophora davidii* and *Rumex hastatus* exhibited coordinated osmotic and antioxidant regulation, whereas *Arthraxon lanceolatus*, *Artemisia vestita*, and *Vitex negundo* var. *microphylla* displayed relatively uncoupled and weaker responses to low temperatures.

At 25°C, *Arthraxon lanceolatus* had higher baseline proline concentrations (approximately 2,000 µg g^−1^ fresh weight [FW]) and soluble protein (approximately mg g^−1^ FW) than *Sophora davidii* and *Rumex hastatus* (proline approximately 1,500 µg g^−1^ FW; soluble protein approximately mg g^−1^ FW), whereas all species shared comparable superoxide dismutase activity (20–30 U g^−1^ FW) and soluble sugar levels (5–10 mg g^−1^ FW). With cooling to − 35°C, *Sophora davidii* and *Rumex hastatus* accumulated proline more strongly (exceeding 4,000 µg g^−1^ FW, compared with approximately 3,000 µg g^−1^ FWin *Arthraxon lanceolatus*) and maintained higher superoxide dismutase activity (approximately 65 and 60 U g^−1^ FW at − 25°C, respectively, vs. approximately 40 U g^−1^ FW in *Arthraxon lanceolatus* at − 35°C). Notably, *Sophora davidii* and *Rumex hastatus* increased soluble protein to approximately 3.5 and 3.0 mg g^−1^ FW, respectively, by − 25°C, whereas *Arthraxon lanceolatus* showed a decline. Soluble sugars in *Sophora davidii* and *Rumex hastatus* peaked at approximately 35 and 30 mg g^−1^ FW, respectively, compared with approximately 20 mg g^−1^ FW in *Arthraxon lanceolatus*, with *Sophora davidii* retaining approximately 30 mg g^−1^ FW even at − 35°C.

Collectively, *Sophora davidii* and *Rumex hastatus* exhibited coordinated enhancement of osmolytes (proline, soluble protein, soluble sugars) and superoxide dismutase activity, presumably mitigating cold-induced cellular damage and maintaining homeostasis. In contrast, *Arthraxon lanceolatus* showed sluggish antioxidant defense and weakened osmolyte accumulation under severe cold. These findings confirm that synchronized osmotic adjustment and antioxidant regulation underpin divergent cold tolerance among native species of the Lancang River dry–hot valley, highlighting the adaptive role of these traits in coping with winter chilling.

#### 3.4.3 Chlorophyll pigments

**Figure 5:**
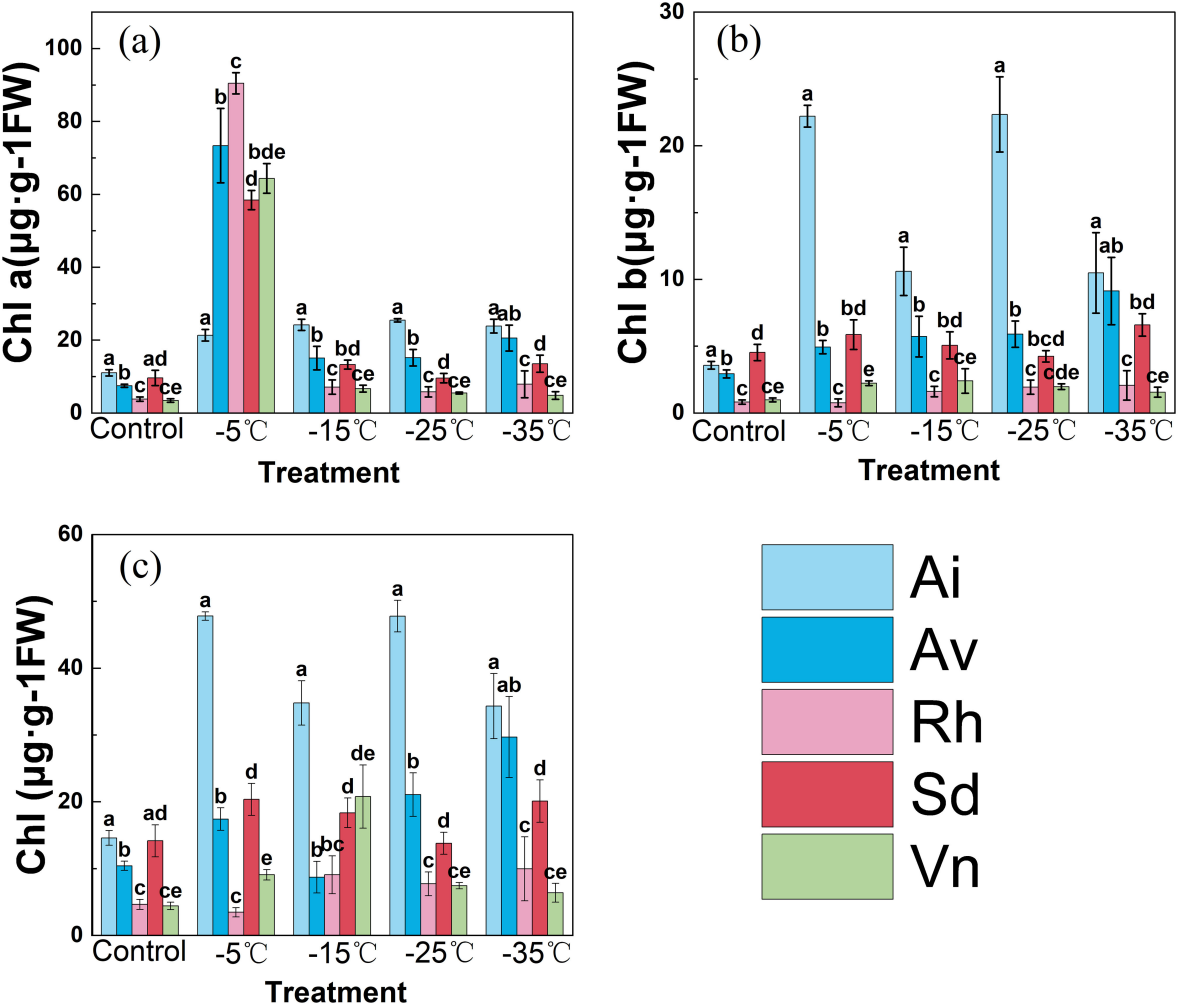
**(A)** Chlorophyll a; **(B)** Chlorophyll b; **(C)** Total chlorophyll contents under each temperature treatment. Effects of low−temperature stress on chlorophyll pigments in five native species. Data are means ± SD (*n*=3). Different lowercase letters above the bars indicate significant differences among species at the same temperature (one−way ANOVA, Duncan’s test, *p*<0.05).

Low temperature significantly altered chlorophyll *a*, chlorophyll *b*, and total chlorophyll contents across all species, with pronounced interspecific variation observed at each temperature gradient (**Figure 5**). The cold-sensitive *Arthraxon lanceolatus* exhibited unstable pigment dynamics, whereas the cold-tolerant *Sophora davidii* and *Rumex hastatus* maintained superior chlorophyll retention throughout the cooling process.

At 25°C, *Arthraxon lanceolatus* had a slightly higher chlorophyll *a* content (approximately 10 µg g^−1^ FW) than *Sophora davidii* and *Rumex hastatus*, while chlorophyll *b* levels were comparable (5–6 µg g^−1^ FW) among the three species. With cooling to − 35°C, *Arthraxon lanceolatus’s* chlorophyll *a* rose transiently to approximately 30 µg g^−1^ FW at − 5°C, then plummeted to approximately 5 µg g^−1^ FW, and its chlorophyll *b* steadily declined to approximately 2 µg g^−1^ FW. In contrast, *Sophora davidii* preserved chlorophyll *a* at approximately 15 µg g^−1^ FW and chlorophyll *b* at 5–8 µg g^−1^ FW at − 35°C; *Rumex hastatus* peaked at approximately 35 µg g^−1^ FW chlorophyll *a* at − 15°C and retained approximately 12 µg g^−1^ FW at − 35°C, with stable chlorophyll *b* levels. Total chlorophyll followed similar trends: *Sophora davidii* and *Rumex hastatus* maintained 20–40 µg g^−1^ FW across most temperatures, while *Arthraxon lanceolatus* dropped from approximately 15 to approximately 7 µg g^−1^ FW.

These results confirm that *Sophora davidii* and *Rumex hastatus* tightly regulate chlorophyll metabolism—sustaining biosynthesis and constraining catabolism—to maintain a stable pigment pool for light harvesting. As chlorophyll loss reduces light-harvesting potential capacity and often accompanies damage to the photosynthetic apparatus, the robust chlorophyll homeostasis observed in *Sophora davidii* and *Rumex hastatus* provides a mechanistic explanation for their higher actual quantum yield of Photosystem II and electron transport rate performance, as well as their overall superior cold tolerance compared with *Arthraxon lanceolatus*.

#### 3.4.4 Proline biosynthetic enzyme activity

**Figure 6:**
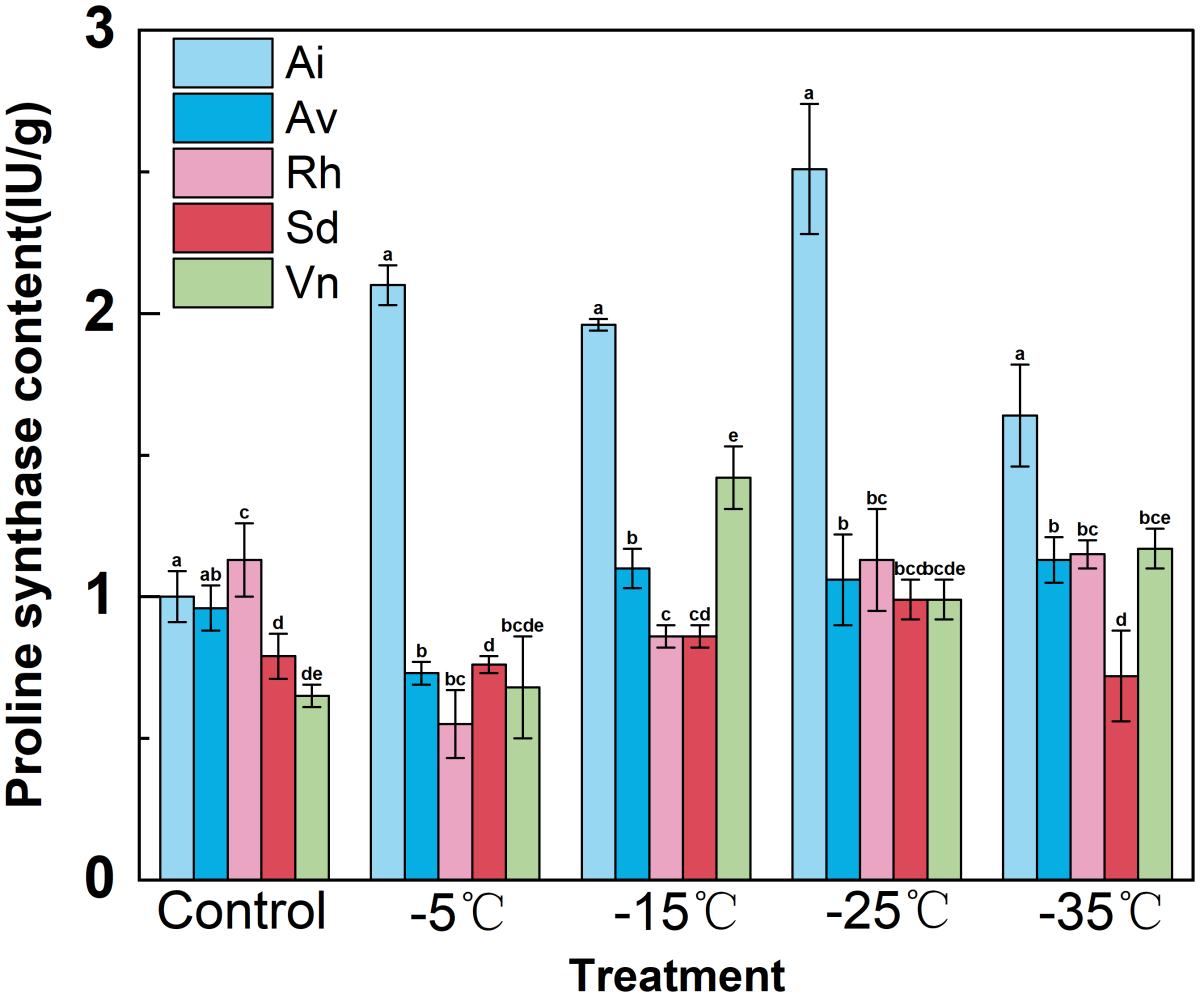
Effects of low−temperature stress on proline synthase activity in leaves of five native species. Data are means ± SD (*n*=3). Different lowercase letters above the bars indicate significant differences among species at the same temperature (one−way ANOVA, Duncan’s test, *p*<0.05).

The activity of Δ^1^-pyrroline-5-carboxylate synthetase—a key proline-biosynthetic enzyme—showed distinct temperature-dependent and species-specific responses among the studied plants (**Figure 6**). Cold-tolerant *Rumex hastatus* and *Sophora davidii* exhibited sustained Δ^1^-pyrroline-5-carboxylate synthetase activation, whereas cold-sensitive species displayed inconsistent or weak enzyme responses.

At 25°C, Δ^1^-pyrroline-5-carboxylate synthetase activity was comparable across all species (0.8–1.0 IU g^−1^), indicating similar baseline proline biosynthetic capacity. With cooling from − 5°C to − 35°C, *Arthraxon lanceolatus* exhibited a “transient upregulation followed by a sharp decline”: Δ^1^-pyrroline-5-carboxylate synthetase surged to approximately 2.5 IU g^−1^ at − 25°C but dropped to approximately 1.5 IU g^−1^ at − 35°C. In contrast, *Rumex hastatus* and *Sophora davidii* maintained graded, persistent activation—*Rumex hastatus’s* Δ^1^-pyrroline-5-carboxylate synthetase increased steadily to approximately 1.2 IU g^−1^, and *Sophora davidii* rose moderately to approximately 0.8 IU g^−1^. *Artemisia vestita* and *Vitex negundo* var. *microphylla* retained low Δ^1^-pyrroline-5-carboxylate synthetase activity (0.5–0.8 IU g^−1^) throughout the temperature gradient.

Collectively, the sustained, moderate activation of Δ^1^-pyrroline-5-carboxylate synthetase in *Rumex hastatus* and *Sophora davidii* supports continuous proline biosynthesis, maintaining osmolyte homeostasis and underpinning their superior cold resilience. In contrast, the transient enzyme activation in *Arthraxon lanceolatus* and the insufficient Δ^1^-pyrroline-5-carboxylate synthetase activity in *Artemisia vestita*/*Vitex negundo* var. *microphylla* fail to sustain effective osmotic adjustment, consistent with these species’ higher susceptibility to cold-induced cellular imbalance.

### 3.5 Integrated evaluation of cold tolerance

#### 3.5.1 Correlation analysis among cold-stress indicators

**Figure 7:**
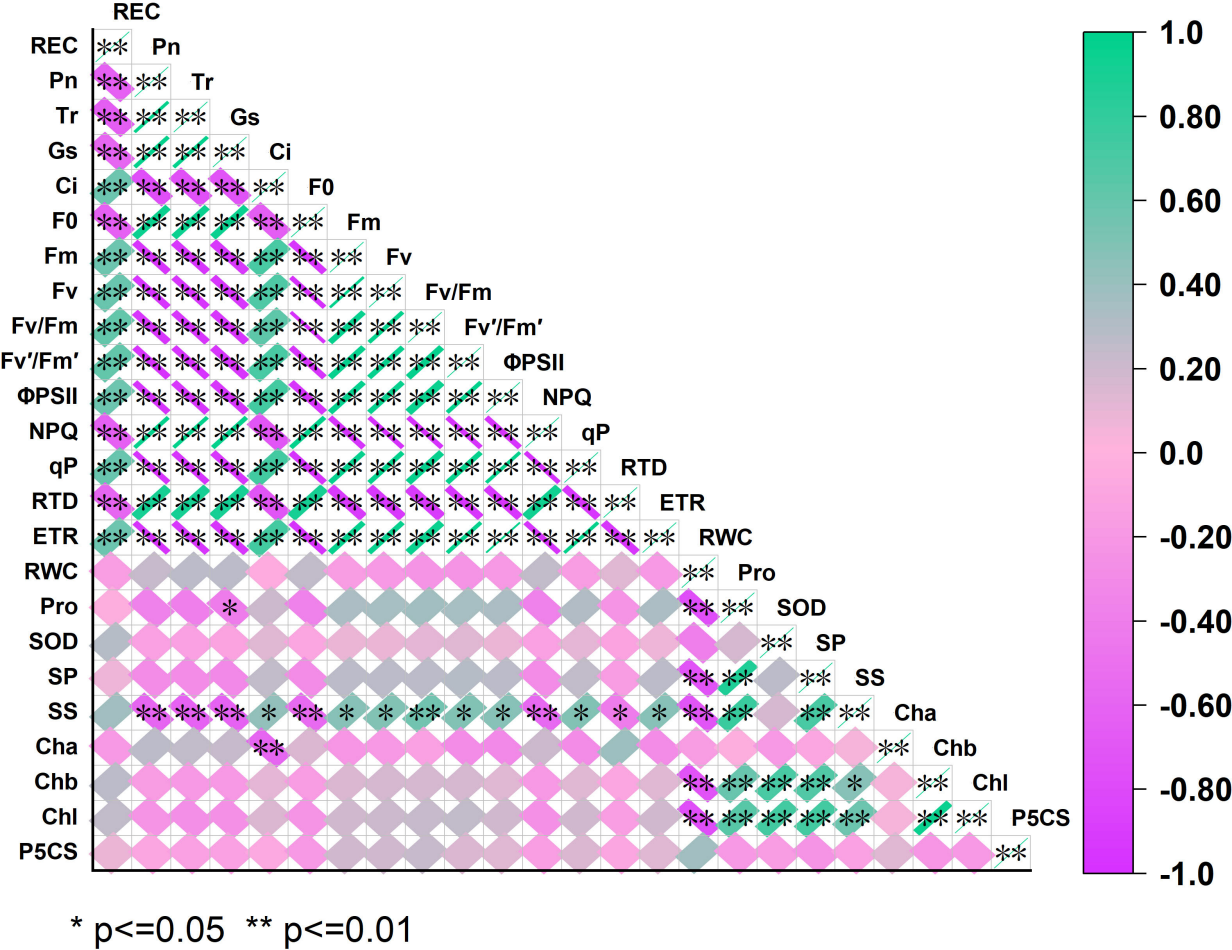
Pearson correlation matrix of physiological and biochemical indices under low−temperature stress. The color gradient represents correlation coefficients (1.0=strong positive; − 1.0=strong negative); ^*^p<0.05 and ^**^p<0.01 denote statistically significant correlations. Indices are defined as follows: Gas exchange and membrane traits: REC, relative electrical conductivity; Pn, net photosynthetic rate); Tr, transpiration rate; Gs, stomatal conductance; Ci, intercellular CO_2_ concentration. PSII fluorescence indices: *F*_0_, basal fluorescence; *F_m_*, maximal fluorescence; *F_v_*, variable fluorescence; *F_v_*/*F_m_*, maximum quantum yield of PSII; *F_v_*′/*F_m_*′, effective quantum yield of PSII; ΦPSII, actual quantum yield of PSII; NPQ, nonphotochemical quenching; qP, photochemical quenching; RTD, regulated thermal dissipation; ETR, electron transport rate. Water and metabolic traits: RWC, relative water content; Pro, proline; SOD, superoxide dismutase; SP, soluble protein; SS, soluble sugars. Chlorophyll and enzyme: Cha, chlorophyll *a*; Chb, chlorophyll *b*; Chl, total chlorophyll; P5CS, Δ^1^-pyrroline-5-carboxylate synthetase.

This Pearson correlation heatmap illustrates associations among 24 physiological indices of dry–hot valley plants under low-temperature stress (**Figure 7**) (color gradient: green=positive correlation, purple=negative correlation; ^*^p<0.05; ^**^p<0.01). Net photosynthetic rate, transpiration rate, and stomatal conductance showed strong positive correlations (^**^p<0.01), whereas intercellular CO_2_ concentration was negatively linked to these gas-exchange traits.

Photosystem II indices—including basal fluorescence, maximal fluorescence, variable fluorescence, maximum quantum yield of Photosystem II, effective quantum yield of Photosystem II, actual quantum yield of Photosystem II, and electron transport rate—correlated positively with net photosynthetic rate, transpiration rate, and stomatal conductance. Nonphotochemical quenching was negatively associated with net photosynthetic rate and maximum quantum yield of Photosystem II, reflecting a trade-off between photochemical efficiency and regulated heat dissipation.

Relative electrolyte leakage, a membrane injury indicator, was strongly negatively correlated with photosynthetic and Photosystem II-related indices (^**^p<0.01), emphasizing the role of membrane integrity in maintaining physiological functions. Osmotic indices (proline, soluble protein, and soluble sugars), the antioxidant trait superoxide dismutase, chlorophyll indices (chlorophyll *a*, chlorophyll *b*, and total chlorophyll), and Δ^1^-pyrroline-5-carboxylate synthetase also exhibited significant internal correlations and links to these core traits.

#### 3.5.2 Principal component analysis of cold-stress indicators and integrated appraisal of cold tolerance

**Figure 8:**
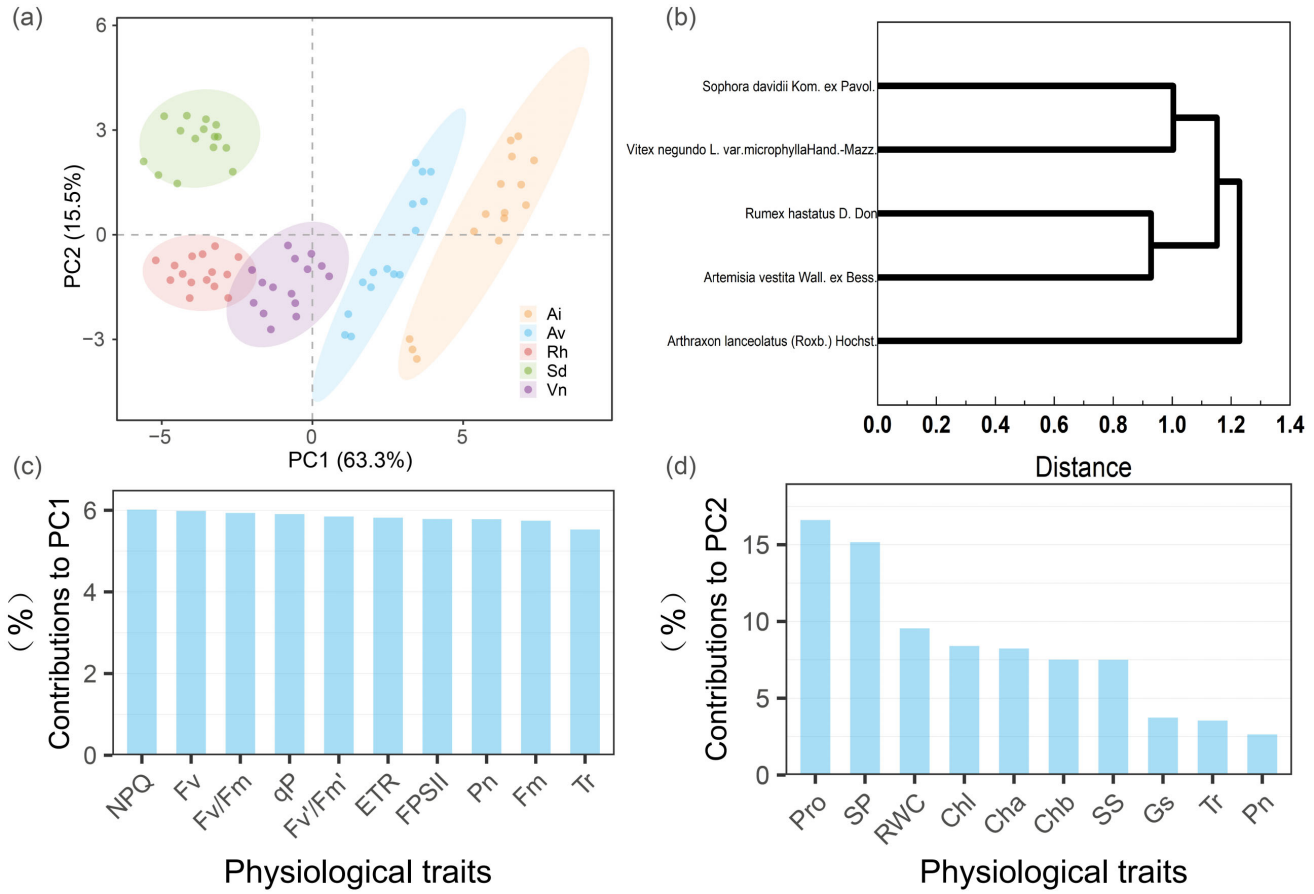
Principal component analysis (PCA) of cold−stress indicators in five native species and hierarchical clustering based on cold−stress indicators. **(a)** Principal component analysis of 24 related indicators for five plants; **(b)** comprehensive clustering analysis of five plants; **(c)** ranking chart of the contributions of each indicator to principal component 1 (top 10); **(d)** ranking of the contributions of each indicator to principal component 2 (top 10). **(a)** Plant abbreviation full names: Ai, *Arthraxon lanceolatus*; Av, *Artemisia vestita*; Rh, *Rumex hastatus*; Sd, *Sophora davidii*; Vn, *Vitex negundo* var. *microphylla*. Species names in the figures are abbreviated accordingly. The indicators in **(c, d)** include: NPQ, nonphotochemical quenching; *F_v_*, variable fluorescence; *F_v_*/*F_m_*, variable fluorescence/maximum fluorescence; qP, Pphotochemical quenching; *F_v_*′/*F_m_*′, variable fluorescence/maximum fluorescence, light-adapted; ETR, electron transport rate; FPSII, quantum yield of Photosystem II photochemistry; Pn, net photosynthetic rate; *F_m_*, maximum fluorescence; Tr, transpiration rate; Pro, proline; SP, soluble protein; RWC, relative water content; Chl, chlorophyll; Cha/Chb, chlorophyll *a*/chlorophyll *b*; SS, soluble sugar; and Gs, stomatal conductance.

Principal component analysis of cold-stress-associated physiological and biochemical traits identified two dominant axes explaining 78.8% of the total variance: the first principal component accounted for 63.3%, and the second for 15.5% (**Figure 8**). Samples from the distinct species *Arthraxon lanceolatus*, *Artemisia vestita*, *Rumex hastatus*, *Sophora davidii*, and *Vitex negundo* var. *microphylla* were clearly segregated along these axes. For example, *Sophora davidii* clustered in the positive direction of the second principal component, while *Arthraxon lanceolatus* was distributed along the positive first principal component, reflecting progressive physiological divergence under increasing cold stress.

The first principal component was defined by opposing Photosystem II photochemical traits (e.g., maximum quantum yield of Photosystem II, actual quantum yield of Photosystem II, electron transport rate, and net photosynthetic rate) and membrane stability indicators (relative electrolyte leakage and intercellular CO_2_ concentration). Accordingly, it represented a “Photosystem II functional efficiency vs. membrane integrity” gradient that encapsulates core photosynthetic responses to cold.

The second principal component, in contrast, represented a gradient of “osmotic/antioxidant compensation vs. tissue hydration”. It was dominated by osmotic adjustment and antioxidant variables (proline, soluble protein, soluble sugars, Δ^1^-pyrroline-5-carboxylate synthetase, and superoxide dismutase) as well as chlorophyll contents (total chlorophyll, chlorophyll *a*, and chlorophyll *b*), in opposition to relative water content. Contribution analyses (right panels) indicated that Photosystem II fluorescence parameters (e.g., nonphotochemical quenching and maximum quantum yield of Photosystem II) and net photosynthetic rate were the major contributors to the first principal component, whereas osmolytes (proline and soluble protein) and relative water content exhibited the strongest loadings on the second principal component. These patterns emphasize that photosynthetic maintenance and metabolic adjustment play distinct, critical roles in shaping cold-stress responses.

To unify these multidimensional traits into a cold-tolerance metric, principal component analysis scores were combined with a membership-function approach to derive a composite cold-tolerance index. Groups with high scores on the first principal component (sustained Photosystem II performance) and moderate-to-high scores on the second principal component (effective osmotic/antioxidant compensation) were classified as cold tolerant, those with low scores on both axes were cold sensitive, and intermediates as moderately tolerant. This cold-tolerance index ranking aligned with semilethal temperature estimates and prior clustering results, confirming that this two-axis framework robustly captures interspecific cold-tolerance variation among native species of the Lancang River dry–hot valley.


$$\begin{array}{l} Y1=0.446X1+0.97X2-0.06X3-0.012X4-0.537X5+0.234X6+0.103X7-0.056X8-0.104X9+0.72X10+0.09X11-0.244X12-0.458X13+0.15X14+0.28X15-0.094X16+0.983X17+\\ 0.974X18+0.989X19+0.6X20+0.031X21+0.554X22+0.846X23-0.169X24 \end{array}$$

$$\begin{array}{l} Y2=0.656X1+0.065X2+0.993X3-0.994X4+0.078X5-0.964X6+0.465X7+0.822X8+0.877X9-0.101X10-0.392X11+0.088X12-0.013X13-0.12X14-0.004X15-0.803X16-\\ 0.15X17-0.009X18-0.091X19-0.161X20+0.478X21-0.001X22+0.028X23+0.408X24 \end{array}$$

$$\begin{array}{l} Y3=-0.516X1-0.036X2+0.099X3+0.042X4-0.766X5+0.019X6-0.011X7-0.303X8-0.379X9+0.013X10+0.829X11-0.1X12+0.869X13-0.94X14+0.892X15-0.588X16+\\ 0.088X17-0.101X18+0.056X19+0.782X20+0.245X21+0.623X22+0.485X23+0.264X24 \end{array}$$

$$\begin{array}{l} Y4=0.323X1-0.232X2-0.013X3-0.102X4-0.344X5-0.122X6+0.879X7+0.48X8+0.276X9+0.686X10+0.388X11-0.961X12+0.186X13+0.283X14+0.354X15+0.009X16+\\ 0.066X17+0.202X18+0.105X19-0.05X20+0.843X21+0.551X22+0.218X23+0.858X24 \end{array}$$

The comprehensive evaluation index was calculated as: *Y*=36.988*Y1 + 30.72*Y2 + 20.749*Y3 + 11.544*Y4.

As shown in **Table 2**, the comprehensive evaluation scores for *Sophora davidii*, *Rumex hastatus*, *Vitex negundo* var. *microphylla*, *Artemisia vestita*, and *Arthraxon lanceolatus* are 0.146, 0.952, 1.716, 3.277, and 2.973, respectively. *Artemisia vestita* > *Arthraxon lanceolatus* > *Vitex negundo* var. *microphylla* > *Rumex hastatus* > *Sophora davidii*.

**Table 2:** Comprehensive evaluation results of principal components.

Plant Name	F1	F2	F3	F4	Comprehensive evaluation score	Ranking
Sd	0.412	0.498	− 0.500	− 0.481	0.146	5
Rh	3.782	− 1.058	− 1.588	1.799	0.952	4
Vn	1.294	1.052	1.952	4.414	1.716	3
Av	6.493	− 0.356	3.097	2.965	3.277	1
Ai	4.177	2.760	0.357	4.384	2.973	2

As shown in **Table 3**, based on the average membership function values of the cold hardiness coefficients for the 24 indicators, the order of cold hardiness from strongest to weakest is as follows: *Artemisia vestita* > *Arthraxon lanceolatus* > *Vitex negundo* var. *microphylla* > *Rumex hastatus* > *Sophora davidii*.

**Table 3:** Average membership function values of cold tolerance coefficients of 24 indicators.

Species	Sd	Rh	Vn	Av	Ai
REC	0.974	0.649	1.000	0.902	0.000
Pn	0.371	0.650	0.000	1.000	0.750
Tr	0.618	0.000	0.411	0.334	1.000
Gs	0.542	1.000	0.686	0.790	0.000
Ci	0.000	0.132	0.530	1.000	0.402
*F*_0_	0.574	0.000	0.488	0.144	1.000
*F_m_*	0.000	0.361	0.787	0.361	1.000
*F_v_*	0.209	0.178	0.423	0.000	1.000
*F_v_*/*F_m_*	0.383	0.212	0.360	0.000	1.000
*F_v_*′/*F_m_*′	0.000	0.878	0.689	1.000	0.956
ΦPSII	0.000	0.130	0.914	1.000	0.046
NPQ	1.000	0.273	0.000	0.279	0.155
qP	0.514	0.000	1.000	0.660	0.254
RTD	0.177	1.000	0.214	0.000	0.618
ETR	0.094	0.000	0.731	1.000	0.398
RWC	0.305	1.000	0.351	0.185	0.000
Chl *a*	0.000	0.592	0.018	1.000	0.558
Chl *b*	0.000	0.764	0.082	1.000	0.854
Chl	0.000	0.610	0.035	1.000	0.640
Pro	0.057	0.000	0.206	1.000	0.137
SOD	0.000	0.186	0.931	0.459	1.000
SP	0.000	0.297	0.669	1.000	0.630
SS	0.000	0.270	0.253	1.000	0.560
P5CS	0.000	0.126	1.000	0.317	0.847
*R* ^2^	0.242	0.388	0.491	0.643	0.575
Ranking	5	4	3	1	2

The abbreviations of the plant physiological and biochemical indices correspond to their full names: *REC*, relative electrical conductivity; *Pn*, net photosynthetic rate; *Tr*, transpiration rate; *Gs*, stomatal conductance; *Ci*, intercellular CO_2_ concentration; *F_0_*, basal fluorescence; *F_m_*, maximal fluorescence; *F_v_*, variable fluorescence; *F_v_/F_m_*, maximum quantum yield of Photosystem II; *F_v_*′/*F_m_*′, effective quantum yield of Photosystem II; *ΦPSII*, actual quantum yield of Photosystem II; *NPQ*, nonphotochemical quenching; *qP*, photochemical quenching; *RTD*, regulated thermal dissipation; *ETR*, electron transport rate; *RWC*, relative water content; *Chl a*, chlorophyll *a*; *Chl b*, chlorophyll *b*; *Chl*, total chlorophyll; *Pro*, proline; *SOD*, superoxide dismutase; *SP*, soluble protein; *SS*, soluble sugars; *P5CS*, Δ^1^-pyrroline-5-carboxylate synthetase.

Unweighted pair group method with arithmetic mean clustering was performed using Euclidean distances derived from core cold-tolerance indices, including relative electrolyte leakage; Photosystem II photochemical efficiencies (maximum quantum yield of Photosystem II, actual quantum yield of Photosystem II), osmolytes (proline, soluble sugars), and antioxidant capacity (superoxide dismutase). The five native plant species were clustered into three distinct groups (**Figure 8**), with stable boundaries at a Euclidean distance coefficient of ~ 1.0, reflecting clear interspecific differences in cold adaptation strategies.

Cluster I included *Sophora davidii* and *Vitex negundo* var. *microphylla*. Despite differences in their absolute lethal temperature for 50% of individuals, these two species shared a “membrane protection/Photosystem II stability” adaptive strategy. At − 15°C, their relative electrolyte leakage increased moderately (≤ ~ 40%), while Photosystem II performance remained high (maximum quantum yield of Photosystem II > 0.6), indicating minimal cold-induced membrane damage and sustained electron transport capacity. The consistent temperature-response patterns of relative electrolyte leakage and Photosystem II metrics across the cold gradient explain their co-clustering.

Cluster II comprised *Rumex hastatus* and *Artemisia vestita*, which adopted a “metabolic compensation” strategy. Progressive cooling triggered substantial proline accumulation (up to ~300% increase at − 5°C to − 15°C) and sustained high superoxide dismutase activities (*Rumex hastatus* ≈ 60 U g^−1^ FW; *Artemisia vestita* ≈ 55 U g^−1^ FW). The coordinated enhancement of osmotic adjustment (via proline and soluble sugars) and reactive oxygen species scavenging (via superoxide dismutase) mitigated cold-induced damage, explaining the close clustering of these species.

Cluster III was a solitary group consisting of *Arthraxon lanceolatus*, characterized by the highest semilethal temperature for 50% of individuals (− 5.10°C) and early cold-induced damage. At − 5°C, *Arthraxon lanceolatus* exhibited relative electrolyte leakage ≥ 80% and maximum quantum yield of Photosystem II<0.5, reflecting severe membrane disruption and impaired Photosystem II function. Poor membrane stability and inadequate metabolic buffering (limited osmolyte accumulation and antioxidant activation) indicated low intrinsic cold tolerance, consistent with its reliance on phenological escape rather than physiological resistance.

Unweighted pair group method with arithmetic mean clustering results corroborated lethal temperature 50% of individuals-based cold tolerance rankings and revealed three distinct cold adaptation pathways: *Sophora davidii* and *Vitex negundo* var. *microphylla* (membrane/Photosystem II stability), *Rumex hastatus* and *Artemisia vestita* (osmolyte-antioxidant compensation), and *Arthraxon lanceolatus* (cold-sensitive phenotype). This pattern aligns with four functional axes identified by principal component analysis, providing a robust basis for selecting cold-tolerant species and guiding ecological restoration in cold-prone areas of the Lancang River dry–hot valley.

## 4 Discussion

### 4.1 Ecological meaning of semilethal temperature and cold-tolerance classes

The semilethal temperature is a critical link between cellular-level damage (e.g., membrane and organelle disruption) and whole-plant survival, ultimately influencing community stability during periods of extreme cold (Murchie and Lawson, 2013). In the present study, lethal temperature 50% values followed a clear gradient: *Sophora davidii* (− 26.87°C)<*Rumex hastatus* (− 25.59°C)<the perennial herb *Artemisia vestita* (− 23.71°C)<< the annual/short-lived herb *Arthraxon lanceolatus* (− 5.10°C). This gradient from woody shrubs to annual herbs aligns with the alpine-gorge concept, which proposes that large diurnal temperature ranges and episodic cold events favor woody, long-lived plants consistent with findings on the natural overwintering cold hardiness of woody seedlings (Wang and Wang, 2020). In contrast, fast-growing life histories rely on phenological avoidance, resulting in inherently lower cold tolerance despite this adaptive strategy (Myers et al., 2000). Notably, the lethal temperature 50% values of *Sophora davidii* and *Rumex hastatus* are far below the historic minimum temperature (~ − 15°C) in the study region, indicating a substantial safety buffer against sudden cold snaps. These species thus hold potential as ecosystem stabilizers under the increasing frequency of extreme cold events.

Cellular injury dynamics further supported this pattern. The relative electrolyte leakage–temperature curves of *Sophora davidii* and *Rumex hastatus* exhibited classical S-shaped trajectories: electrolyte leakage increased slowly between − 5°C and − 15°C, followed by a steep rise around species-specific transition points (≈ − 20°C for *Sophora davidii*). This resulted in better-fitted logistic models (*R*^2^ ≈ 0.692 and 0.668, respectively) compared with the more cold-sensitive *Arthraxon lanceolatus* and *Vitex negundo* var. *microphylla* (*R*^2^ ≈ 0.562). These response patterns indicate a concentrated threshold (low variance) in highly cold-tolerant taxa, whereas sensitive taxa show a flatter and more variable response (Neri et al., 2024). To refine cold tolerance classification, we integrated lethal temperature 50% with Photosystem II photochemical performance (maximum quantum yield of Photosystem II, actual quantum yield of Photosystem II) and osmotic/antioxidant markers, establishing an evidential chain linking membrane stability → energy conversion efficiency → metabolic defense capacity. Shrubs exhibited principal component loadings consistent with their lethal temperature 50% rankings, highlighting the dominant role of membrane and photochemical stability in cold tolerance. In contrast, herbaceous species showed weaker coordination among these traits, which aligns with their greater reliance on phenological avoidance (Pearce, 2001).

Integrating all these physiological and ecological metrics, we classify *Sophora davidii* and *Rumex hastatus* as highly cold tolerant, *Artemisia vestita* as moderately cold tolerant, and *Arthraxon lanceolatus* (along with *Vitex negundo* var. *microphylla*) as cold sensitive. This classification reflects selective pressures imposed by the dry–hot valley’s microclimate and topographic heterogeneity and provides operational thresholds for prioritizing species in ecological restoration initiatives.

### 4.2 Coordinated roles of membrane stability and oxidative–osmotic regulation

The plasma membrane is the first and most direct target of freezing injury: the transition of membrane water to an amorphous state, along with the accompanying loss of semipermeability, leads to increased relative electrolyte leakage and accumulation of malondialdehyde—an indicator of structural damage and lipid oxidation (Qian et al., 2024). In our results, *Sophora davidii* maintained consistently lower relative electrolyte leakage (approximately 70% even at − 35°C) than the other species, whereas *Arthraxon lanceolatus* and *Rumex hastatus* reached near 100% at just − 5°C. These findings suggest species-specific thresholds for preserving membrane integrity.

Mechanistically, cold induces bursts of reactive oxygen species as electron transport is hindered. If antioxidant defenses (superoxide dismutase, peroxidase, catalase, ascorbate peroxidase) respond slowly, oxidative damage escalates; however, if they respond rapidly, damage remains limited (Ruban, 2016). In *Sophora davidii*, we observed a combination of low relative electrolyte leakage/low malondialdehyde levels and high superoxide dismutase activity, indicating effective reactive oxygen species scavenging that protects membrane lipids. This can be summarized as: “reactive oxygen species clearance → lipid peroxidation control → membrane intactness”.

Osmotic adjustment provides a parallel line of defense. Accumulation of proline and soluble sugars helps stabilize membranes and protein hydration shells, buffering against dehydration- and volume-induced permeability changes. In *Sophora davidii*, proline content rose significantly at − 35°C and soluble sugars at − 25°C, coinciding with its low relative electrolyte leakage values. This indicates that when osmolytes and antioxidants act in concert, they create a cellular environment that is both osmotically stable and protected from reactive oxygen species damage (Foyer and Noctor, 2020; Jahed and Van den Ende, 2023; Tarkowski and Van den Ende, 2015). The metabolic changes observed, such as proline accumulation, are likely regulated by the CBF-COR pathway, a key hub for cold signaling (Shi et al., 2018), and cold acclimation processes (Xin and Browse, 2000). Additionally, Shi et al. (2016) highlighted the critical role of abscisic acid (ABA) in mediating these cold resistance signaling networks.

An important aspect of osmotic tolerance is the baseline membrane lipid composition. For example, *Artemisia vestita* accumulated proline levels similar to *Sophora davidii* at − 35°C, but its relative electrolyte leakage remained relatively high (approximately 85%–95%), suggesting that membrane composition (e.g., fatty acid unsaturation, sterol and sphingolipid content) can limit the benefits of proline/soluble sugar accumulation (Szabados and Savouré, 2010; Shomo et al., 2024). Insufficient membrane unsaturation might prevent osmolytes from fully preserving membrane fluidity through phase transitions.

Photosystem II function is also tightly linked to membrane status. Membrane destabilization and reactive oxygen species can depress both the maximum quantum yield of Photosystem II and the actual quantum yield of Photosystem II, reflecting impaired energy transfer and electron flow (Thalhammer et al., 2014a; Tarkowski and Van den Ende, 2015). Indeed, species with high relative electrolyte leakage (indicating greater membrane injury) under cold conditions showed stronger declines in Photosystem II performance. Using both membrane-level (relative electrolyte leakage, malondialdehyde) and function-level (maximum quantum yield of Photosystem II, actual quantum yield of Photosystem II) indicators together can improve cold-tolerance assessment by reducing the bias or “blind spots” inherent in any single metric (Thomashow, 1999a; Thalhammer et al., 2014b).

Overall, our findings suggest that maintaining winter membrane stability depends on a triad of factors: robust antioxidant capacity, effective osmotic adjustment, and favorable membrane lipid traits. Species that excel in all three dimensions (e.g., *Sophora davidii*) exhibit system-level cold tolerance, which underscores the value of multilevel evaluation rather than reliance on a single endpoint.

### 4.3 Divergent PSII and chlorophyll–antenna responses and their links to cold tolerance

Photosystem II functionality can be maintained under cold conditions if its quantum efficiencies (maximum quantum yield of Photosystem II, actual quantum yield of Photosystem II) and electron transport rate are preserved; in that case, carbon assimilation remains a controlled loss rather than dropping to zero as temperature decreases (Thomashow, 1999b). We observed that at − 35°C, *Sophora davidii* still retained a maximum quantum yield of Photosystem II of ≈ 0.410 and an electron transport rate of ≈ 18 µmol m^−2^ s^−1^, higher than the values observed for *Arthraxon lanceolatus*. This aligns with the general trend that cold lowers maximum Photosystem II efficiency across all species but widens interspecific differences in light utilization capacity (Verhoeven, 2014). Such a physiological buffer helps explain why the most tolerant species can sustain some photosynthetic activity at the coldest temperature.

Mechanistically, *Sophora davidii* benefits from multiple protective strategies acting in concert. First, on the supply side, it preserves chlorophyll content and antenna stability more effectively than sensitive species. *Sophora davidii* maintained higher chlorophyll *a* and relatively stable chlorophyll *b* levels across the temperature gradient, indicating that light harvesting remained largely intact even as membranes stiffened. Cold-tolerant species likely favor the chlorophyll synthesis–degradation balance (e.g., by sustaining Mg-chelatase activity and suppressing chlorophyll catabolic pathways) (Wang, 2020a).

Second, on the allocation side, *Sophora davidii* appears to partition energy between photochemistry and heat dissipation more optimally. Although nonphotochemical quenching at − 35°C was similarly high in both *Sophora davidii* and *Arthraxon lanceolatus*, *Sophora davidii* achieved a higher actual quantum yield of Photosystem II, implying that it maintained more reaction centers open and functional. In other words, *Sophora davidii* could dissipate excess energy while still using some for photochemistry, whereas *Arthraxon lanceolatus* likely exceeded its photochemical capacity and had to rely entirely on nonphotochemical quenching. A faster relaxation of nonphotochemical quenching (especially dynamic and sustained components) and limited chronic photoinhibition support continued electron flow during stress (Wang, 2007; Wang, 2020; Wei et al., 2022).

Third, on the repair side, *Sophora davidii* may maintain more effective Photosystem II repair cycles. Cold and reactive oxygen species together slow D1 protein turnover in Photosystem II, hampering the repair of photodamaged reaction centers. However, *Sophora davidii* likely mitigates this by promptly re-initiating net D1 synthesis once the acute stress passes. By returning more quickly to a net repair state, it limits the buildup of inactive Photosystem II centers despite a lower absolute maximum quantum yield of Photosystem II at − 35°C.

In addition to these Photosystem II-focused strategies, a sink-side buffer contributes to *Sophora davidii’s* tolerance. We found that *Sophora davidii* maintained higher stomatal conductance and substantial intercellular CO_2_ concentration (≥ 250 µmol mol^−1^) up to − 25°C, suggesting that it kept stomata partially open and internal CO_2_ available when other species had nearly closed stomata at similar temperatures. This likely helped *Sophora davidii* avoid excessive photoinhibition by providing a continued (if reduced) CO_2_ sink for absorbed energy.

Overall, *Sophora davidii* integrates (i) pigment/antenna stability, (ii) efficient nonphotochemical quenching and the actual quantum yield of Photosystem II balancing, (iii) rapid Photosystem II repair, and (iv) moderate stomatal closure to endure extreme cold. Preacclimation of certain traits (e.g., constitutively higher antioxidant levels, flexible nonphotochemical quenching response, cyclic electron flow around photosystem I) could further enhance these protections (Yamori et al., 2020). Our findings for *Sophora davidii* exemplify a “prestabilize → actively control → rapidly repair” strategy that underpins its superior cold tolerance compared to the other species.

### 4.4 Performance of the multi-index framework and divergent cold-tolerance strategies

Cold injury involves a complex interplay of membrane integrity, photochemical efficiency, and metabolic regulation, so no single measurement can fully capture “tolerance”. We found chlorophyll fluorescence to be a particularly useful functional indicator of Photosystem II status that, when combined with other photoinhibition diagnostic indicators (Yang et al., 2020; Yang et al., 2022), provided a strong phenotypic signal of stress. Recent advances in determining fluorescence parameters without dark adaptation (Xia et al., 2023) further validate the utility of these metrics in stress assessment. To synthesize the multidimensional data without redundancy or multicollinearity, we performed principal component analysis, which yielded composite axes with objective weightings (Yang et al., 2002). In our analysis, the first principal component (explaining 60.6% of the variance) was associated with high maximum quantum yield of Photosystem II, actual quantum yield of Photosystem II, electron transport rate, and low relative electrolyte leakage, whereas the second principal component (19.2%) captured variation in proline, soluble sugars, superoxide dismutase, and relative water content. This confirms that Photosystem II efficiency, together with membrane stability, forms the primary axis differentiating these species’ cold responses, with osmotic and antioxidant capacities representing an essential secondary axis. Traits such as relative electrolyte leakage and lethal temperature 50% define absolute limits of membrane stability (Yong et al., 2024), while nonphotochemical quenching (the photoprotective “safety valve” under light during cold) also loaded significantly on the first principal component (Yuan et al., 2024). Osmolyte accumulation and superoxide dismutase activity, defining a metabolic–hydration axis, loaded strongly on the second principal component (Zhang et al., 2019). By combining these indices, the integrated analysis minimizes the blind spots of any single measure and provides a robust multicriteria assessment of cold tolerance.

Using the principal component analysis outputs, we computed a membership function-based comprehensive score for each species (**Table 2**). Interestingly, the ranking by composite score (*Artemisia vestita* > *Arthraxon lanceolatus* > *Vitex negundo* var. *microphylla* > *Rumex hastatus* > *Sophora davidii*) differed from the lethal temperature 50%-based ranking (which was essentially the reverse). The composite scoring, which weighted all factors more evenly, gave *Artemisia vestita* and even *Arthraxon lanceolatus* an advantage due to certain strong compensatory traits (e.g., high nonphotochemical quenching or relative water content bursts), whereas *Sophora davidii* and *Rumex hastatus* scored lower because some of their traits (like nonphotochemical quenching, relative water content) were only moderate despite excellent membrane/Photosystem II stability. By contrast, lethal temperature 50% and relative electrolyte leakage emphasize inherent freeze tolerance and clearly identify *Sophora davidii* and *Rumex hastatus* as top performers.

This divergence suggests that our integrated index captures a different aspect of “cold-hardiness”—one that highlights species with strong acute stress responses (even if they cannot survive the very lowest temperatures). In practical terms, this means that species choice for restoration might depend on the expected cold stress regime. At high-risk sites (where winter extremes regularly approach the cold limits), species with inherently low lethal temperature 50% and superior membrane stability (*Sophora davidii*, *Rumex hastatus*) are indispensable. At moderate-risk sites (where severe freezes are rarer or milder), species with high composite scores (e.g., *Artemisia vestita*, *Vitex negundo* var. *microphylla*)—which may not endure the absolute lowest temperatures but recover quickly from moderate chills—can be included to enhance community resilience.

Finally, our hierarchical clustering analysis (**Figure 8b**) synthesized all indices into three distinct groups corresponding to high, moderate, and low cold tolerance, aligning well with life-history traits. Cluster I contained *Sophora davidii* and *Rumex hastatus* (long-lived shrub/perennial with high tolerance), cluster II grouped *Vitex negundo* var. *microphylla* and *Artemisia vestita* (shrub and perennial with intermediate tolerance), and cluster III consisted of *Arthraxon lanceolatus* (short-lived grass with very low tolerance). This reinforces that woody or long-lived species tend toward a “stress-tolerance” strategy of maintaining membrane and photosynthetic stability (our principal component analysis-first principal component axis), whereas short-lived herbs lean on a “fast-response” strategy of metabolic compensation and avoidance (principal component analysis-second principal component axis). Recognizing these divergent strategies provides a more nuanced understanding of cold tolerance: some species survive by enduring the cold (tolerance type I: *Sophora davidii*, *Rumex hastatus*), others by escaping or quickly compensating for it (tolerance type II: *Arthraxon lanceolatus*, *Artemisia vestita*, etc.). Our multi-index framework successfully captured these differences and can be transferred to cold-tolerance evaluations in other dry–hot valley ecosystems.

### 4.5 Limitations and future work

This study provides empirical baseline cold tolerances for dry–hot valley flora but has three limitations.

#### 4.5.1 Artificial cooling rate

We used a relatively rapid cooling rate of 5°C h^−1^, faster than natural temperature declines of approximately 1°C–2°C h^−1^; therefore, some cold injury may be overestimated. Field overwintering trials are needed to track growth, survival, and physiological responses under natural cold conditions, thereby validating the results obtained from controlled-environment chambers.

#### 4.5.2 Hormonal control

Abscisic acid (ABA), a key stress hormone, promotes stress adaptation by inducing cold-responsive pathways (e.g., CBF-COR) and slowing growth, whereas gibberellins (GA) generally stimulate growth partly by triggering DELLA protein degradation. ABA and GA often act antagonistically during stress acclimation. Accordingly, ABA/GA signaling may contribute to the interspecific differences observed in this study. However, we did not measure hormone contents or the expression of associated genes, so this mechanism remains to be tested in future work.

#### 4.5.3 Root responses

Roots are major cold sensors, and their osmotic and antioxidant capacities influence shoot performance. We focused only on leaves, so the findings cannot be fully extrapolated to whole plants. Future studies should incorporate root traits, belowground rhizosphere microorganisms, and root–shoot linkages to construct a comprehensive cold-resistance framework encompassing the entire plant.

From a holistic perspective, combining multilevel and multi-indicator analyses—linking membrane physics, photochemistry, metabolism, and regulation—is the optimal approach to characterize the cold resistance of native species in the Lancang dry–hot valley. This approach can provide practical guidelines for restoration planning in climatically extreme environments.

## 5 Conclusions

The integrative assessment of five native species revealed clear interspecific differences in cold tolerance, with *Sophora davidii* and *Rumex hastatus* showing the strongest tolerance and *Arthraxon lanceolatus* the weakest. Across metrics, cold-tolerant species maintained lower membrane injury, higher photosynthetic performance, and more coordinated osmotic adjustment and antioxidant protection under severe cold. These findings provide a practical set of traits and candidate species to support cold-resistance breeding and ecological restoration in the Lancang River dry–hot valleys.

## Data Availability

The raw data supporting the conclusions of this article will be made available by the authors, without undue reservation.
