# Supplementary figures and images for: Cold tolerance of native plants in the Lancang River dry–hot valley: an integrative physiological–biochemical assessment with implications for cold-resistance breeding
Source: Front Plant Sci. 2026 Jan 27;16:1724940. doi: 10.3389/fpls.2025.1724940 (PMC12887594; doi:10.3389/fpls.2025.1724940)

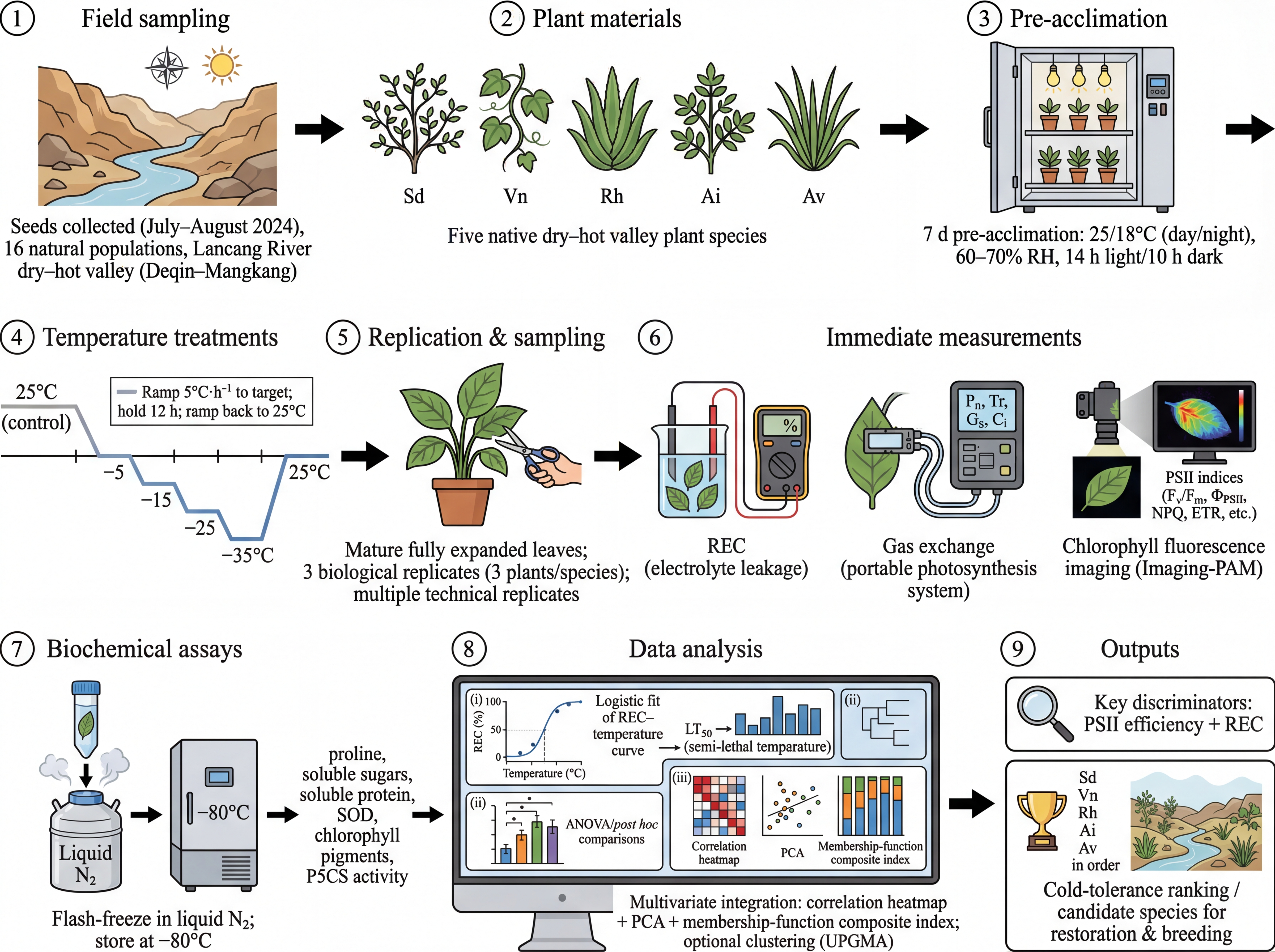

Supplement: Supplementary file 1 [file SupplementaryFile1.jpeg]

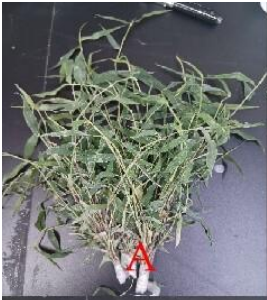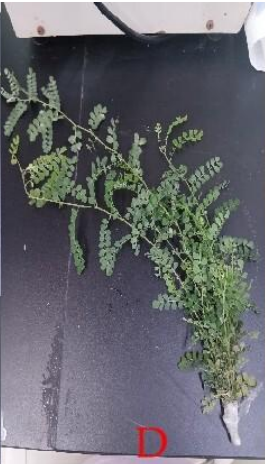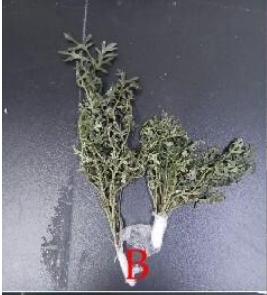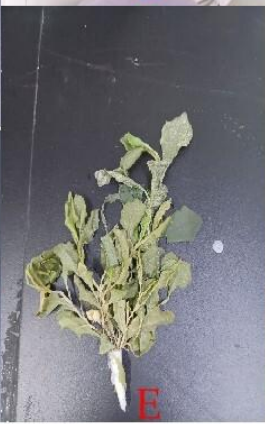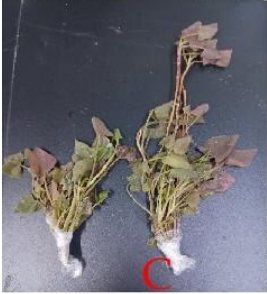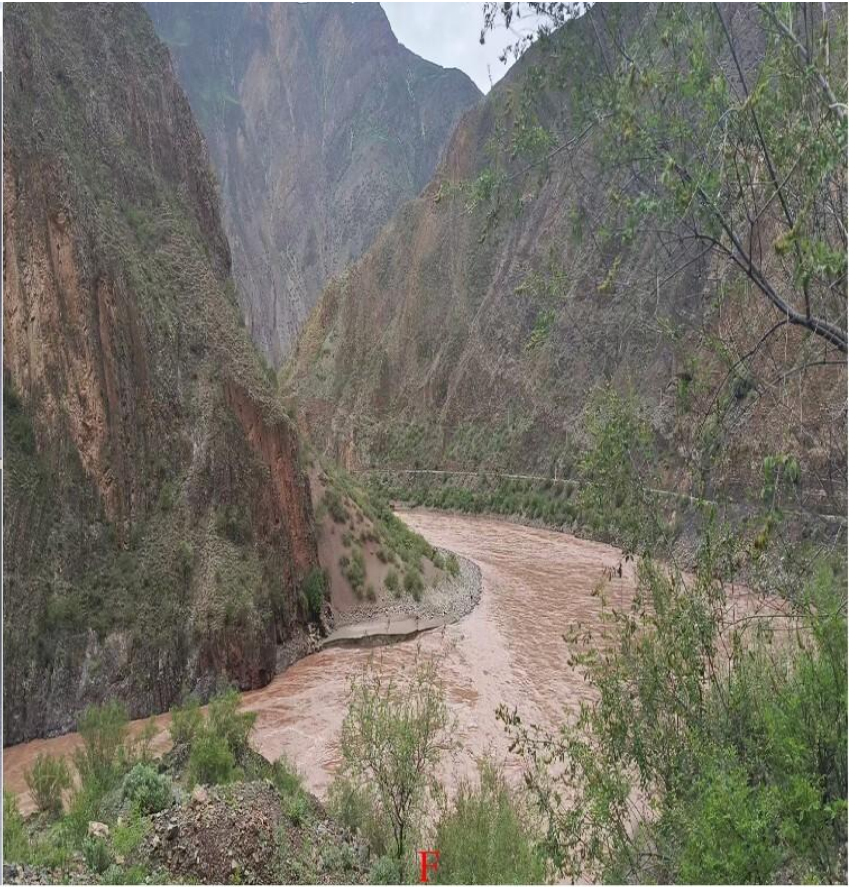

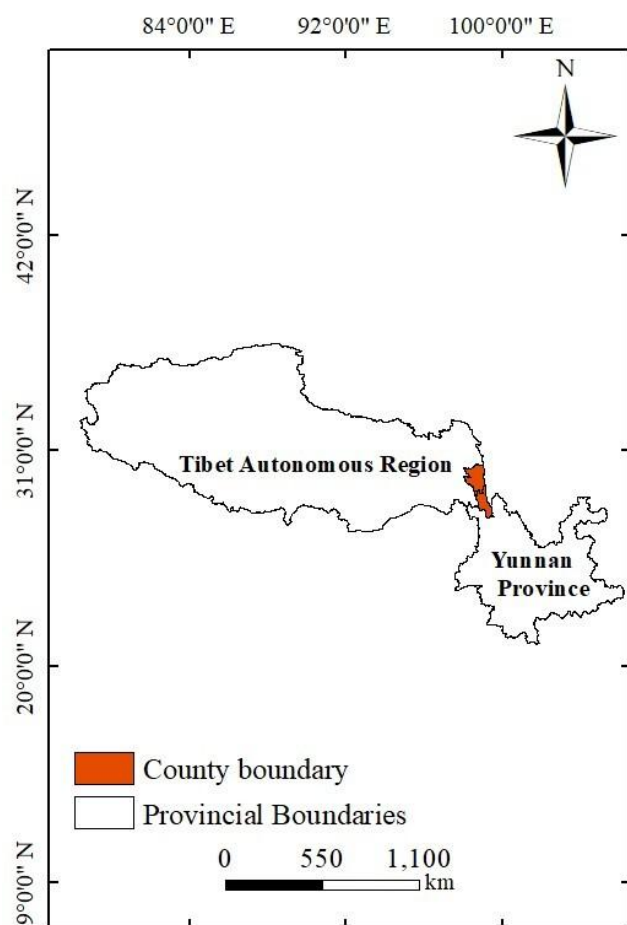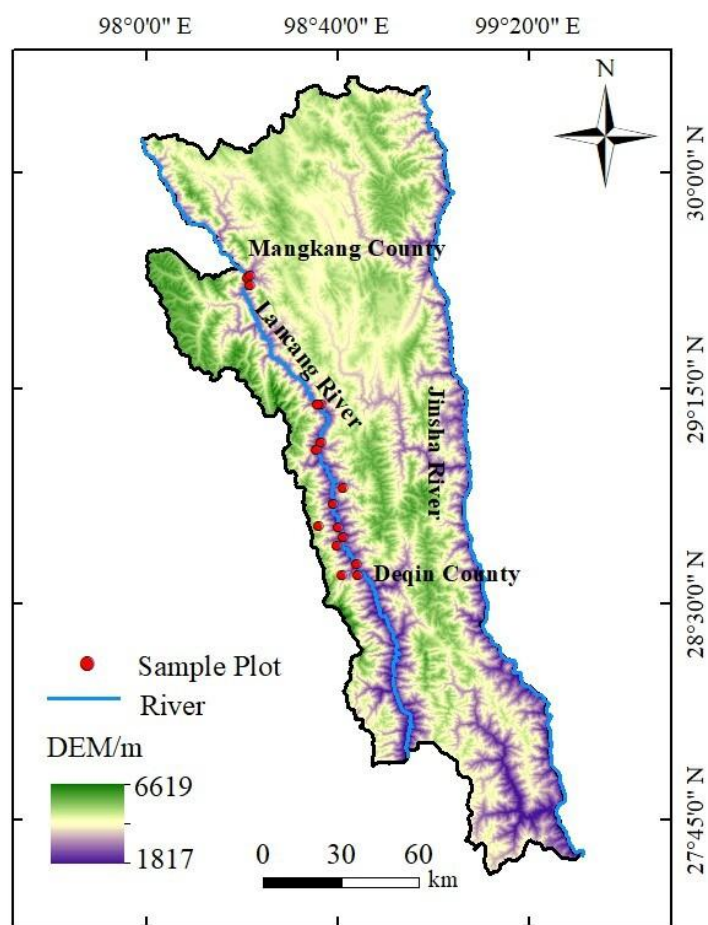

Supplement: Supplementary file 4 [file DataSheet1.pdf]

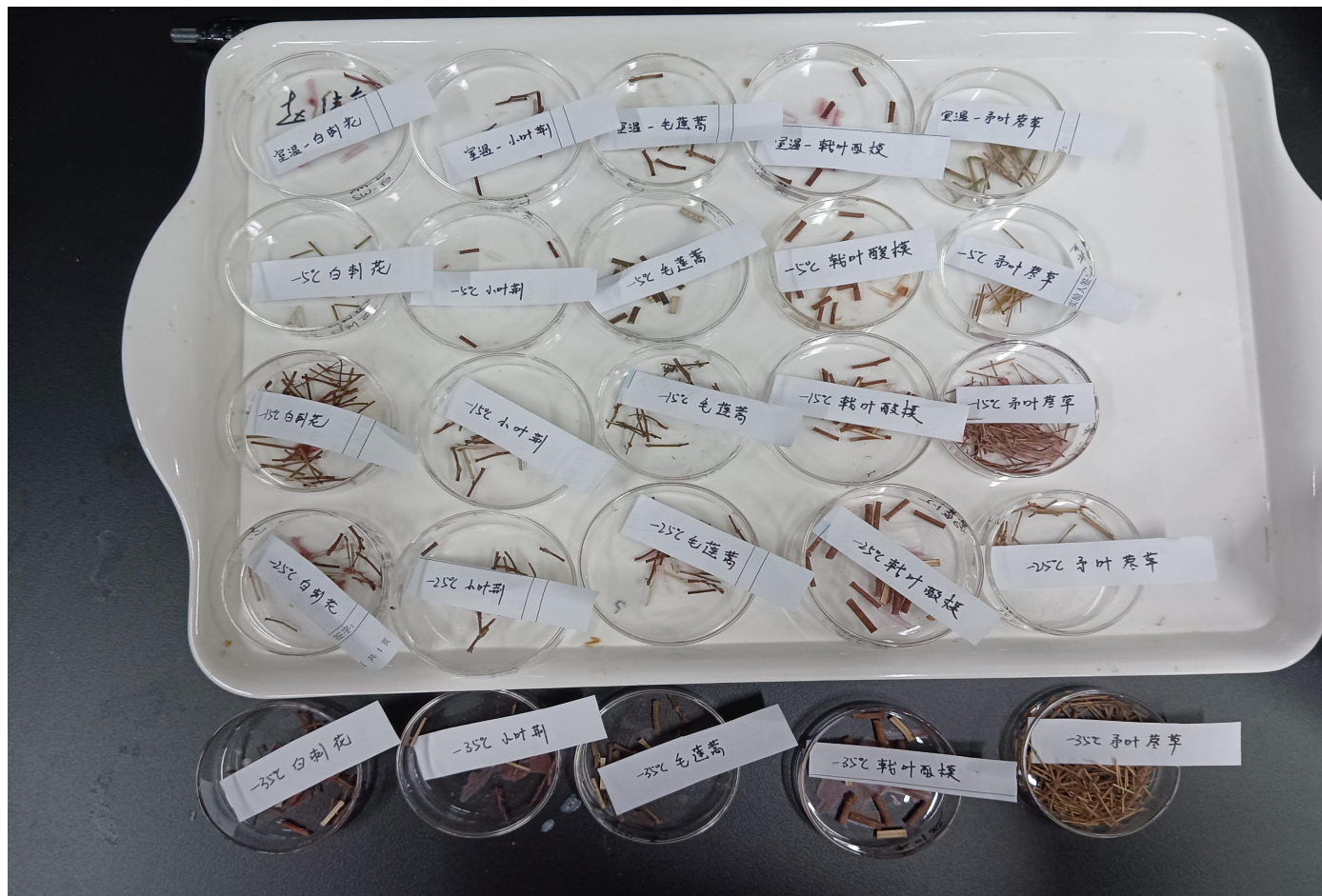

Total sample processing photos

Supplement: Supplementary file 5 [file DataSheet2.pdf]
