# Supplementary material for: Cold tolerance of native plants in the Lancang River dry–hot valley: an integrative physiological–biochemical assessment with implications for cold-resistance breeding
Source: Front Plant Sci. 2026 Jan 27;16:1724940. doi: 10.3389/fpls.2025.1724940 (PMC12887594; doi:10.3389/fpls.2025.1724940)
Supplement: Supplementary file 2 [file Table1.docx]

**Supplementary Table S1**

**TABLE**  Determination of half lethal temperature of plant leaves under low temperature stress.

| **Species** | **Logistic Equation** | **R^2^** | **LT_50_/℃** |
| --- | --- | --- | --- |
| *Arthraxon lanceolatus* (Roxb.) Hochst. | $y=\frac{100}{1+1.58\cdot e^{-0.0892\cdot x}}$ | 0.829 | -5.10 |
| *Artemisia vestita* Wall. ex Bess. | $y=\frac{100}{1+2.05\cdot e^{-0.0303\cdot x}}$ | 0.738 | -23.71 |
| *Rumex hastatus* D. Don | $y=\frac{100}{1+0.127\cdot e^{-0.0808\cdot x}}$ | 0.721 | -25.59 |
| *Sophora davidii* Kom. ex Pavol. | $y=\frac{100}{1+1.99\cdot e^{-0.0257\cdot x}}$ | 0.855 | -26.87 |
| *Vitex negundo* L. var. *microphylla* Hand*.*-Mazz. | $y=\frac{100}{1+0.286\cdot e^{-0.0547\cdot x}}$ | 0.931 | -22.87 |
