# Supplementary material for: Cold tolerance of native plants in the Lancang River dry–hot valley: an integrative physiological–biochemical assessment with implications for cold-resistance breeding
Source: Front Plant Sci. 2026 Jan 27;16:1724940. doi: 10.3389/fpls.2025.1724940 (PMC12887594; doi:10.3389/fpls.2025.1724940)
Supplement: Supplementary file 3 [file Table2.docx]

**Supplementary Table S2**

**TABLE**  Effects of low temperature stress on chlorophyll fluorescence parameters of different plant species.

| **Temperature (°C)** | **Species** | **Chlorophyll fluorescence parameter index** | | | | | | | | | |
| --- | --- | --- | --- | --- | --- | --- | --- | --- | --- | --- | --- |
|  |  | **F0** | **Fm** | **Fv** | **Fv/Fm** | **Fv'/Fm'** | **ΦPSII** | **NPQ** | **qP** | **RTD** | **ETR** |
| 25 | AI | 291.7±14.1 a | 1142.1±57.2 b | 0.493±0.033 a | 0.744±0.022 a | 0.493±0.033 a | 0.371±0.04 a | 0.98±0.15 b | 0.759±0.048 a | 0.588±0.066 b | 78.1±8.9 a |
| 25 | Av | 301.5±20.9 a | 1128.6±40.4 c | 0.457±0.026 b | 0.733±0.021 b | 0.457±0.026 b | 0.321±0.049 b | 1.05±0.08 a | 0.695±0.077 b | 0.627±0.059 a | 66.3±10.8 b |
| 25 | Rh | 269.6±19.0 b | 1168.3±52.5 b | 0.531±0.024 a | 0.769±0.02 a | 0.531±0.024 a | 0.398±0.036 a | 0.91±0.09 b | 0.757±0.052 a | 0.601±0.032 a | 81.7±8.4 a |
| 25 | Sd | 290.6±16.4 a | 1206.7±72.0 a | 0.536±0.027 a | 0.758±0.026 a | 0.536±0.027 a | 0.42±0.038 a | 0.87±0.09 c | 0.767±0.059 a | 0.593±0.043 a | 86.7±7.5 a |
| 25 | Vn | 295.1±18.5 a | 1128.7±45.2 c | 0.502±0.033 a | 0.739±0.012 a | 0.502±0.033 a | 0.365±0.034 a | 0.88±0.12 c | 0.727±0.025 b | 0.591±0.086 a | 77.0±4.7 a |
| -5 | AI | 332.4±17.3 a | 1134.8±80.6 a | 0.476±0.011 a | 0.706±0.022 c | 0.476±0.011 a | 0.305±0.029 b | 1.25±0.15 a | 0.649±0.055 a | 0.639±0.022 a | 63.1±6.5 b |
| -5 | Av | 305.7±18.3 b | 1057.0±50.4 b | 0.463±0.033 b | 0.71±0.027 b | 0.463±0.033 b | 0.298±0.031 b | 1.29±0.11 a | 0.628±0.035 b | 0.651±0.051 a | 64.2±7.0 b |
| -5 | Rh | 312.5±15.1 a | 1125.3±82.0 a | 0.458±0.056 b | 0.721±0.032 b | 0.458±0.056 b | 0.339±0.052 a | 1.25±0.11 a | 0.743±0.065 a | 0.609±0.055 b | 70.7±9.1 a |
| -5 | Sd | 310.5±14.1 a | 1177.5±40.6 a | 0.483±0.019 a | 0.736±0.015 a | 0.483±0.019 a | 0.339±0.03 a | 1.22±0.1 b | 0.704±0.027 a | 0.607±0.05 b | 71.2±7.2 a |
| -5 | Vn | 320.8±12.3 a | 1105.6±39.0 a | 0.446±0.021 b | 0.71±0.011 b | 0.446±0.021 b | 0.307±0.054 b | 1.3±0.09 a | 0.677±0.08 a | 0.616±0.078 b | 66.3±14.7 a |
| -15 | AI | 363.9±6.4 a | 973.5±42.1 b | 0.411±0.022 a | 0.626±0.016 b | 0.411±0.022 a | 0.239±0.017 a | 1.87±0.08 b | 0.583±0.013 a | 0.674±0.039 a | 50.2±5.4 a |
| -15 | Av | 359.1±25.1 a | 982.2±26.5 b | 0.367±0.041 b | 0.634±0.032 b | 0.367±0.041 b | 0.204±0.03 a | 1.83±0.12 b | 0.545±0.024 b | 0.702±0.077 a | 40.3±6.7 b |
| -15 | Rh | 348.3±17.2 b | 1015.2±38.4 a | 0.431±0.02 a | 0.657±0.019 a | 0.431±0.02 a | 0.248±0.02 a | 1.88±0.14 b | 0.589±0.072 a | 0.589±0.039 b | 53.2±3.7 a |
| -15 | Sd | 359.1±14.9 a | 991.2±31.9 a | 0.419±0.026 a | 0.637±0.022 b | 0.419±0.026 a | 0.234±0.034 a | 1.9±0.11 a | 0.557±0.055 b | 0.682±0.028 a | 50.8±6.2 a |
| -15 | Vn | 366.5±18.6 a | 966.3±70.6 b | 0.396±0.03 a | 0.619±0.029 c | 0.396±0.03 a | 0.196±0.029 b | 1.95±0.13 a | 0.521±0.044 c | 0.661±0.067 a | 42.1±5.8 b |
| -25 | AI | 399.5±21.4 a | 869.2±47.7 b | 0.33±0.013 a | 0.539±0.044 b | 0.33±0.013 a | 0.144±0.012 a | 2.63±0.1 a | 0.428±0.042 a | 0.712±0.031 b | 30.0±2.9 a |
| -25 | Av | 415.1±31.8 a | 892.9±23.0 a | 0.287±0.03 b | 0.534±0.045 b | 0.287±0.03 b | 0.116±0.019 b | 2.66±0.09 a | 0.401±0.036 b | 0.749±0.03 a | 25.3±4.3 b |
| -25 | Rh | 406.1±22.5 a | 943.3±36.8 a | 0.32±0.022 a | 0.569±0.028 a | 0.32±0.022 a | 0.134±0.016 a | 2.68±0.08 a | 0.418±0.049 a | 0.753±0.06 a | 28.9±5.2 a |
| -25 | Sd | 379.4±25.7 b | 902.7±50.5 a | 0.333±0.03 a | 0.579±0.028 a | 0.333±0.03 a | 0.138±0.016 a | 2.57±0.08 b | 0.425±0.031 a | 0.73±0.041 a | 29.2±2.8 a |
| -25 | Vn | 400.7±33.4 a | 832.3±46.8 b | 0.309±0.027 a | 0.518±0.04 b | 0.309±0.027 a | 0.129±0.007 a | 2.62±0.09 b | 0.417±0.039 a | 0.71±0.04 b | 26.5±1.8 b |
| -35 | AI | 446.9±12.8 c | 790.6±35.0 a | 0.259±0.018 b | 0.434±0.026 a | 0.259±0.018 b | 0.079±0.009 b | 3.37±0.08 a | 0.337±0.033 a | 0.774±0.05 a | 17.3±3.9 a |
| -35 | Av | 470.4±7.0 b | 786.4±63.2 a | 0.263±0.008 a | 0.399±0.042 a | 0.263±0.008 a | 0.086±0.018 a | 3.35±0.09 a | 0.321±0.039 b | 0.742±0.039 b | 17.6±4.7 a |
| -35 | Rh | 450.7±39.1 c | 843.9±38.0 a | 0.271±0.015 a | 0.465±0.057 a | 0.271±0.015 a | 0.092±0.013 a | 3.35±0.1 a | 0.351±0.026 a | 0.742±0.066 b | 17.1±2.5 a |
| -35 | Sd | 468.3±23.6 b | 797.7±42.0 a | 0.263±0.019 a | 0.41±0.063 a | 0.263±0.019 a | 0.09±0.014 a | 3.35±0.1 a | 0.353±0.032 a | 0.752±0.046 a | 18.3±3.7 a |
| -35 | Vn | 488.1±33.3 a | 760.7±18.0 b | 0.259±0.028 b | 0.358±0.052 b | 0.259±0.028 b | 0.088±0.023 a | 3.36±0.15 a | 0.34±0.029 a | 0.748±0.042 a | 17.1±5.9 a |

Note: All results in this table are expressed as mean ± standard deviation. Different lowercase letters (a, b, c, etc.) above the values indicate significant differences among species at the same temperature (one-way analysis of variance (ANOVA) followed by Duncan’s multiple range test, P < 0.05). The letters in the table correspond to the following species: Ai represents *Arthraxon lanceolatus* (Roxb.) Hochst., Vn represents *Vitex negundo* L. var. microphylla Hand.-Mazz., Rh represents *Rumex hastatus* D. Don, Sd represents *Sophora davidii* Kom. ex Pavol., and Av represents *Artemisia vestita* Wall. ex Bess.
