# Supplementary material for: Cold tolerance of native plants in the Lancang River dry–hot valley: an integrative physiological–biochemical assessment with implications for cold-resistance breeding
Source: Front Plant Sci. 2026 Jan 27;16:1724940. doi: 10.3389/fpls.2025.1724940 (PMC12887594; doi:10.3389/fpls.2025.1724940)
Supplement: Supplementary file 6 [file DataSheet3.pdf]

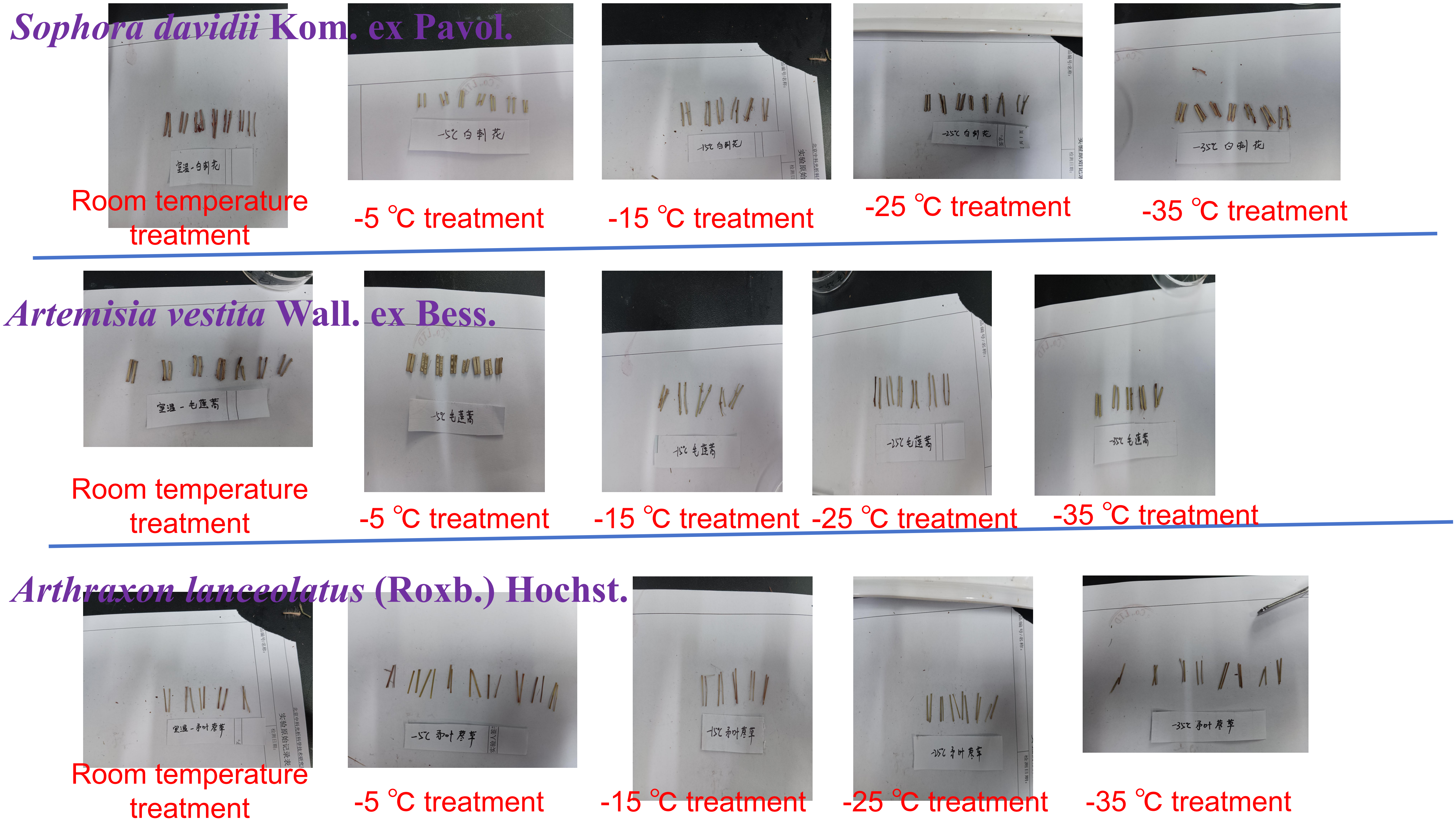
Sophora davidii Kom. ex Pavol.
Room temperature treatment
-5 °C treatment
-15 °C treatment
-25 °C treatment
-35 °C treatment
Artemisia vestita Wall. ex Bess.
Room temperature treatment
-5 °C treatment
-15 °C treatment
-25 °C treatment
-35 °C treatment
Arthraxon lanceolatus (Roxb.) Hochst.
Room temperature treatment
-5 °C treatment
-15 °C treatment
-25 °C treatment
-35 °C treatment

Rumex hastatus D. Don

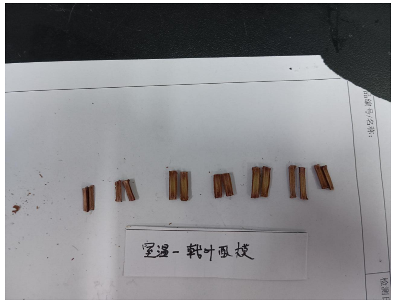

Room temperature treatment

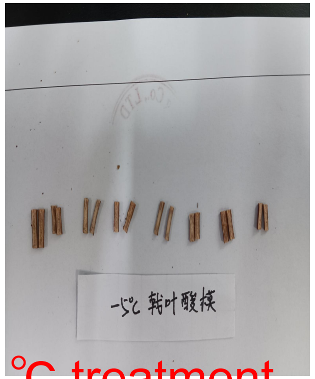

-5 ℃ treatment

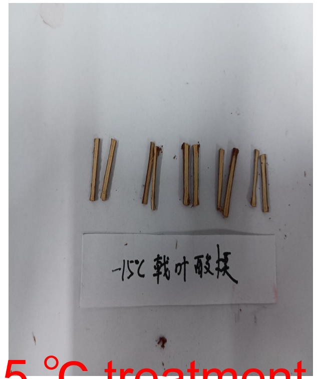

-15 ℃ treatment

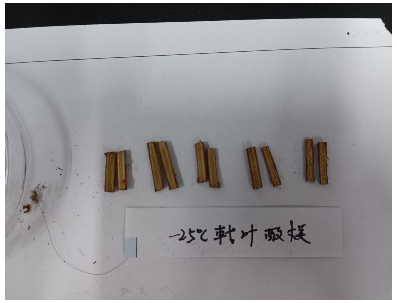

-25 ℃ treatment

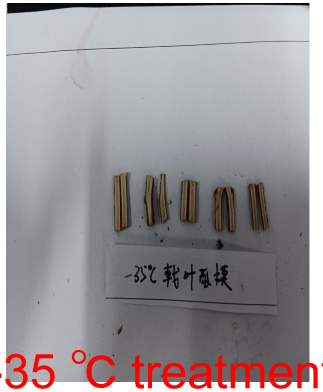

-35 ℃ treatment

VitexnegundoL. var.*microphyllaHand.*-Mazz.

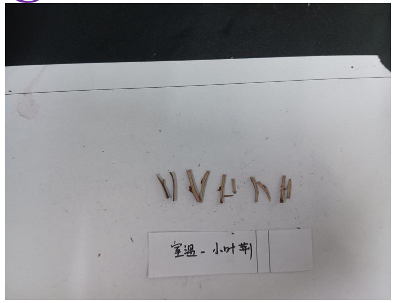

Room temperature treatment

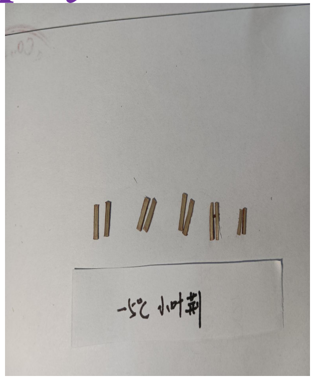

-5 ℃ treatment

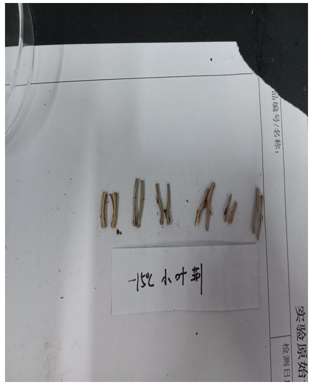

-15 ℃ treatment

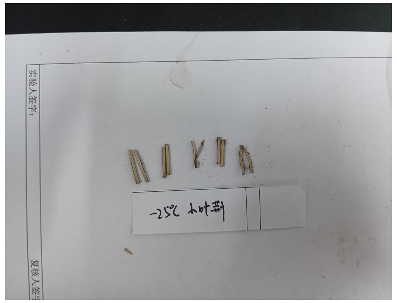

-25 ℃ treatment

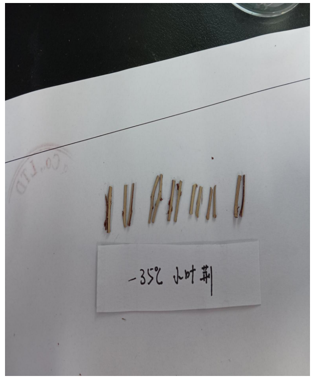

-35 ℃ treatment
