# Supplementary material for: Cold tolerance of native plants in the Lancang River dry–hot valley: an integrative physiological–biochemical assessment with implications for cold-resistance breeding
Source: Front Plant Sci. 2026 Jan 27;16:1724940. doi: 10.3389/fpls.2025.1724940 (PMC12887594; doi:10.3389/fpls.2025.1724940)
Supplement: Supplementary file 7 [file DataSheet4.pdf]

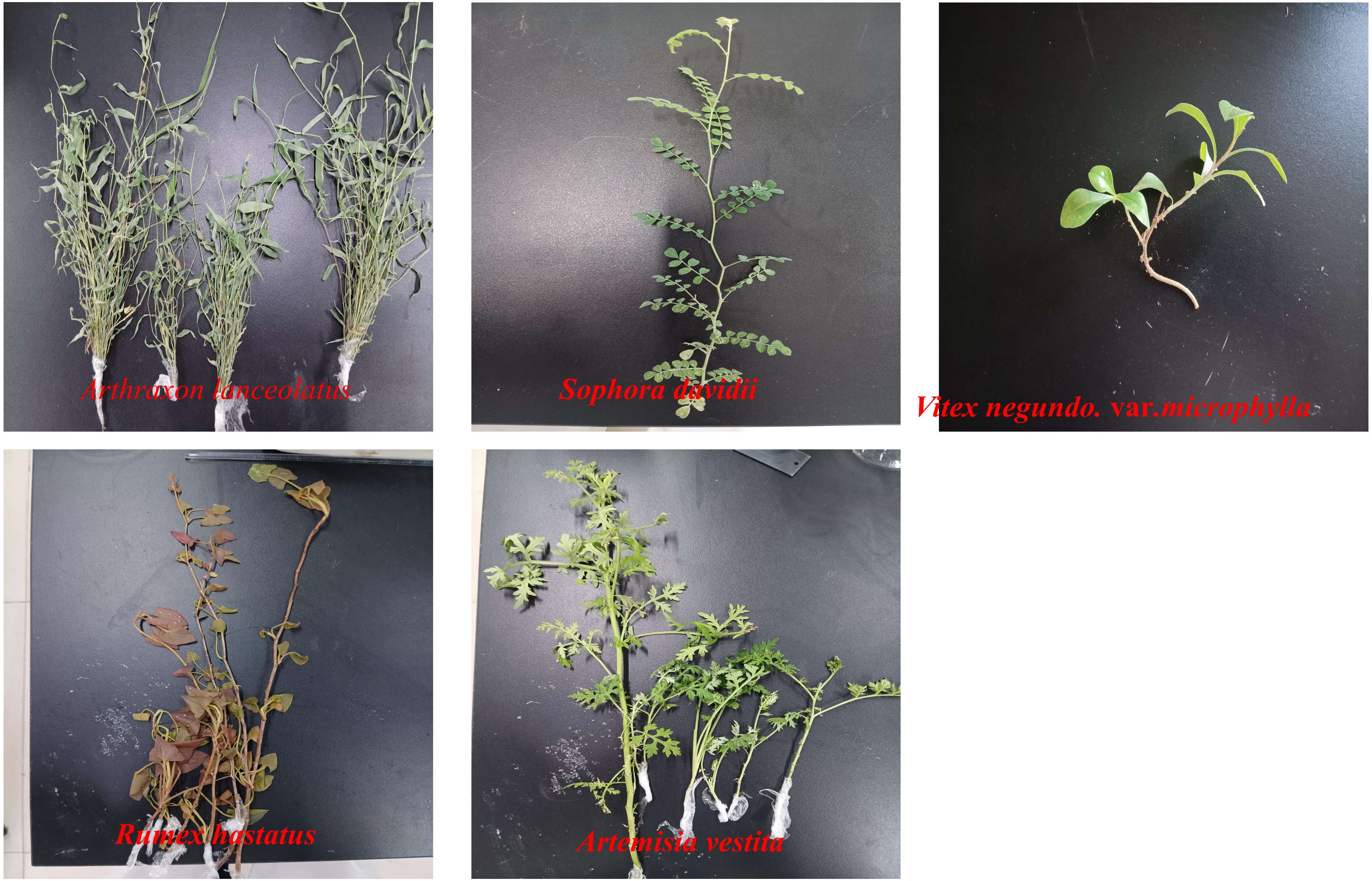

Photos taken during the experimental process of -5 ℃ temperature gradient treatment

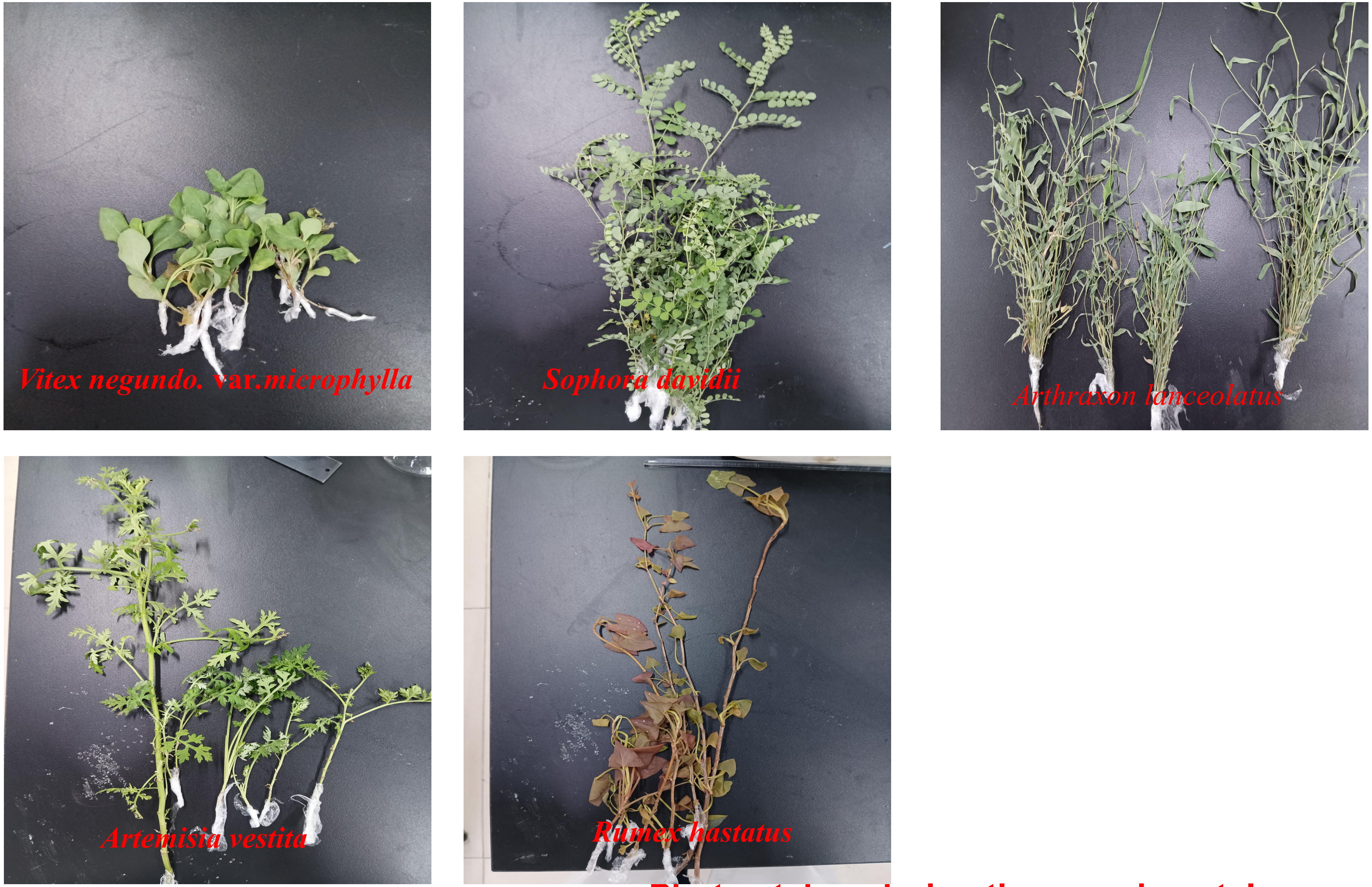

Photos taken during the experimental process of -15 ℃ temperature gradient treatment

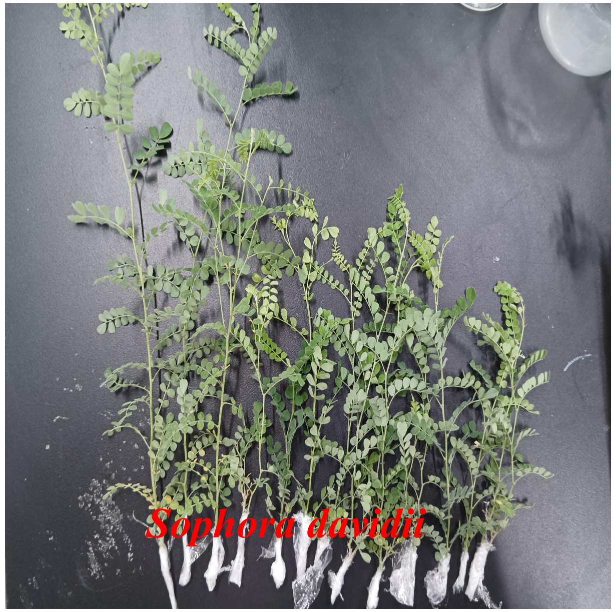

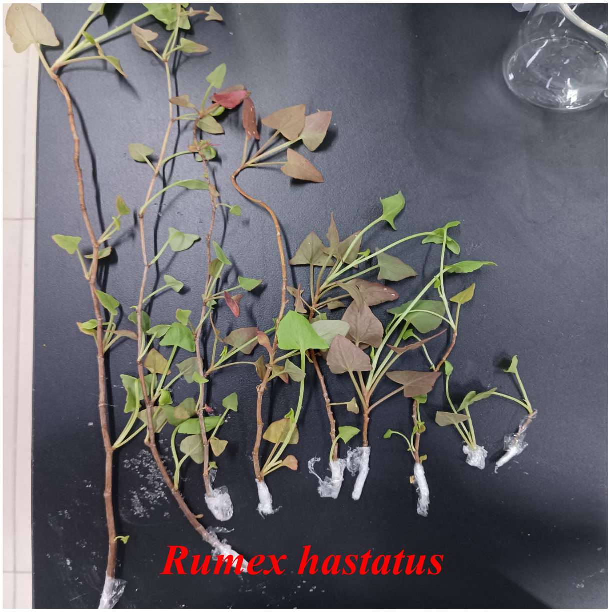

Arthraxon lanceolatus

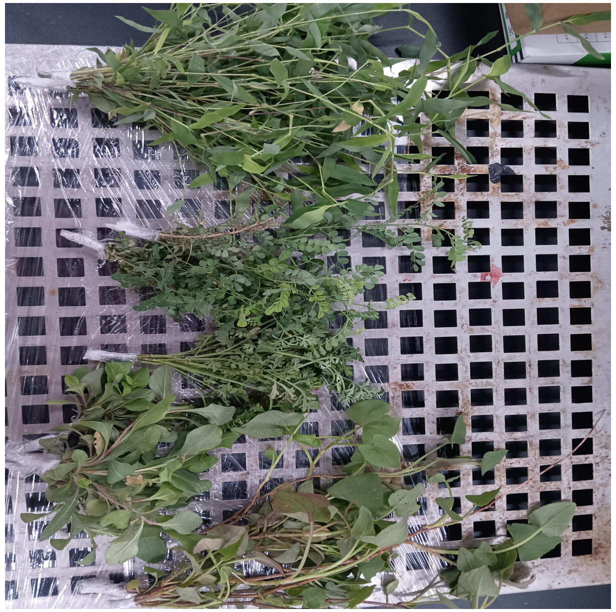

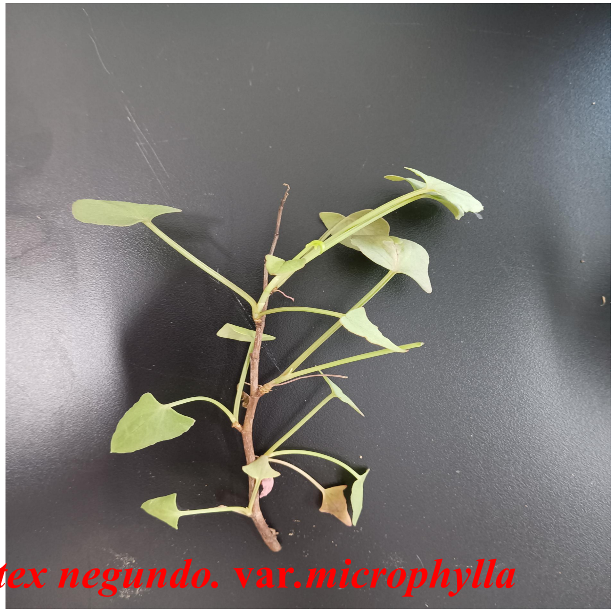

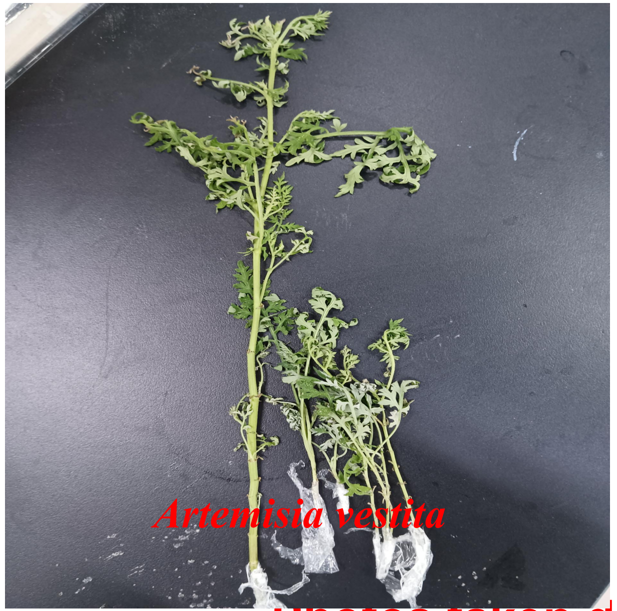

Photos taken during the experimental process of -25 ℃ temperature gradient treatment

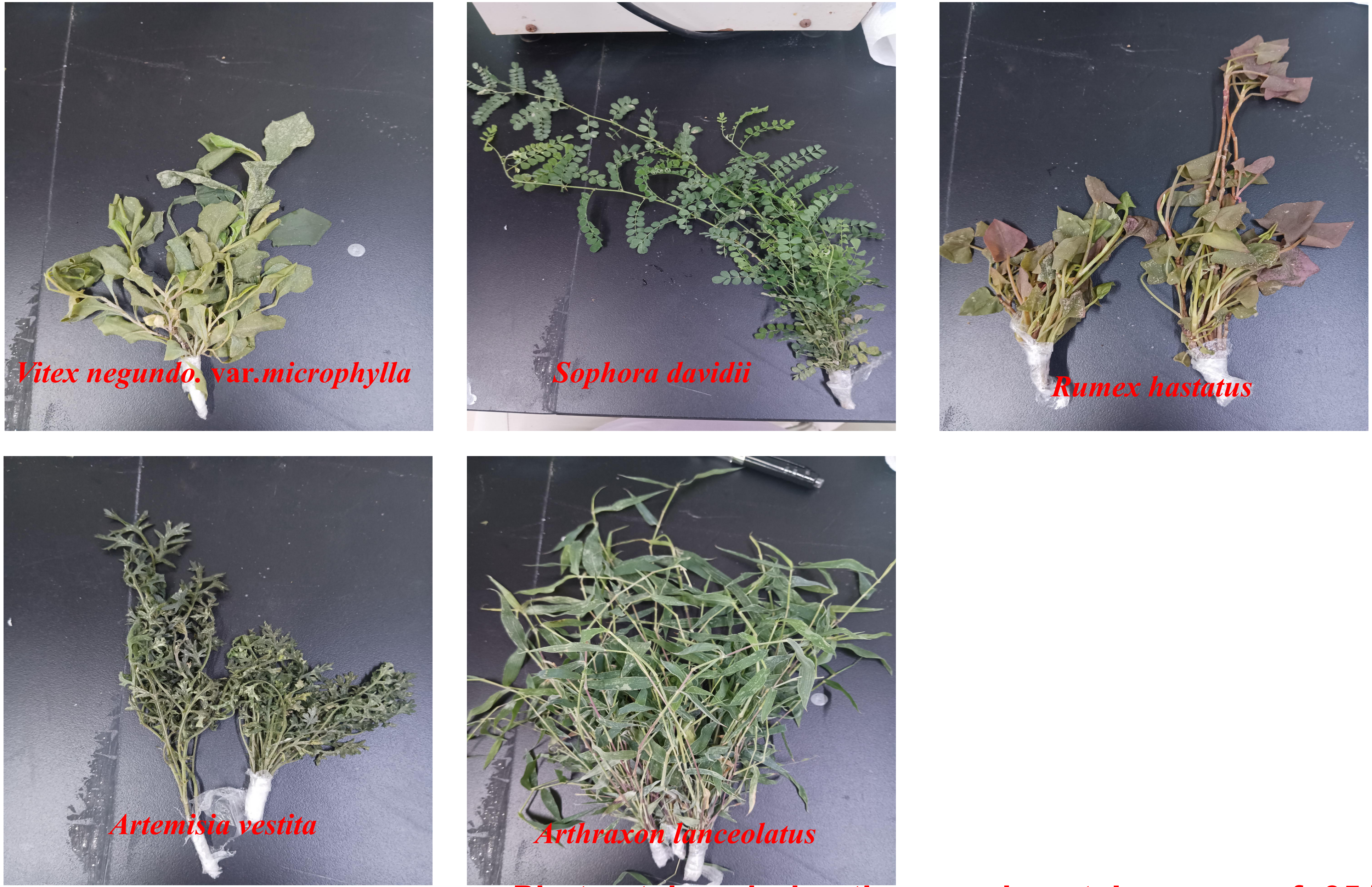

Photos taken during the experimental process of -35 ℃ temperature gradient treatment
